# Real-time monitoring of excess mortality under a new endemic regime

**DOI:** 10.2807/1560-7917.ES.2025.30.25.2400753

**Published:** 2025-06-26

**Authors:** Sasikiran Kandula, Birgitte F de Blasio, Marissa LeBlanc

**Affiliations:** 1 Norwegian Institute of Public Health, Oslo, Norway; 2 Oslo Centre for Biostatistics and Epidemiology, University of Oslo, Oslo, Norway

**Keywords:** mortality surveillance, COVID-19, endemicity, generalised additive models, break points

## Abstract

**BACKGROUND:**

Monitoring of mortality to identify trends and detect deviations from normal levels is an essential part of routine surveillance. In many European countries, disruptions in mortality patterns from the COVID-19 pandemic have required revisions to expected mortality estimates (and models) in the current endemic phase of SARS-CoV-2.

**AIM:**

To identify essential characteristics for future mortality surveillance and describe two Bayesian methods that satisfy these criteria while being robust to past periods of high COVID-19 mortality. We demonstrate their application in 19 European countries and subnational estimates in the United States, and report measures of model calibration.

**METHODS:**

We used a generalised additive model (GAM) with smoothed spline terms for annual trend and within-year seasonality and a generalised linear model (GLM) with a Serfling component for within-year seasonality and breakpoints to detect trend changes in trend. Both approaches modelled change in population size and group-specific (age and sex) mortality patterns.

**RESULTS:**

Models were well-calibrated and able to estimate national and group-specific mortality before and during the acute COVID-19 pandemic phase. The effect of inclusion of mortality from the acute pandemic period was primarily an increase in uncertainty in expected mortality over the projection period. The GAM approach had better calibration and less variability in bias among countries.

**CONCLUSION:**

Models that can adapt to mortality anomalies seen during the acute COVID-19 pandemic period without a need for adjustments to observational data, or tailoring of model specifications, are feasible. The proposed methods can complement operational national and inter-agency surveillance systems currently used in Europe.

**Key public health message**

**What did you want to address in this study and why?**

The COVID-19 pandemic resulted in high mortality in most European countries. With COVID-19 likely to remain a contributing cause of death, we wanted to identify desirable characteristics of statistical models for real-time surveillance of mortality. We propose methods that do not require exclusion or manipulation of mortality observed during the pandemic years, while accounting for group-specific (e.g. age, sex) mortality patterns before and during the pandemic.

**What have we learnt from this study?**

We propose two models for mortality surveillance that meet the identified criteria and can adapt to periods of exceptionally high mortality, e.g. a pandemic. Using public mortality data from 20 countries, 19 in Europe plus the United States, we demonstrate that the models can potentially be useful in real-time mortality surveillance.

**What are the implications of your findings for public health?**

We believe the proposed models can complement mortality surveillance methods currently in use at inter-agency networks such as the EuroMOMO. Collaterally, the identified characteristics may guide the development of alternative approaches.

## Introduction

Monitoring mortality is a key public health activity. All-cause mortality and cause-specific mortality in the population as a whole and in its subgroups, e.g. age and sex, are often included in routine surveillance reports [1,2]. These reports help identify recent trends and detect deviations from normal mortality levels and are of particular relevance in the event of emergence of new pathogens such as severe acute respiratory syndrome coronavirus 2 (SARS-CoV-2). In fact, excess all-cause mortality was used quite extensively as a comprehensive measure of the COVID-19 pandemic burden [3-7].

Conventionally, excess mortality is defined as an increase above an expected range of mortality, the latter interpreted during recent acute pandemic years (ca 2020–April 2023) as mortality that would have occurred without COVID-19 [8-12]. Following declarations from the World Health Organization (WHO) in May 2023 of the end to COVID-19 as a public health emergency [13], most countries transitioned to a post-acute phase of the pandemic by the end of 2023. However, COVID-19 is likely to continue to levy a mortality toll. For routine mortality surveillance, this period will be unlike the pre-pandemic years when COVID-19 was not a contributing cause of death, and different from the acute pandemic years because of built-up protection in the populations from infections and/or vaccinations since 2020 [14-17]. Definitions of expected all-cause mortality that were in use during the pre-pandemic and acute pandemic periods, and models used to estimate them, may need revision, and are the focus of this study.

Until 2020, the model used by the European Mortality Monitoring initiative (EuroMOMO), a network funded by the WHO and the European Centre for Disease Prevention and Control (ECDC) for real-time mortality monitoring in European countries, calculated expected mortality as deaths likely to occur during an average week in summer (weeks 16–25) or autumn (weeks 37–44) [18] of the most recent 5 years (‘reference period’). During the pandemic years 2020–22, the reference period was fixed between 2015 and 2019 and mortality data from more recent years were not included in training the model as these were considered anomalous [19]. Inclusion of real-time data resumed in spring 2023 with the continued exclusion of a 3-year acute pandemic period. In the United States (US), the Centers for Disease Control and Prevention (CDC) published real-time excess mortality estimates between April 2020 and September 2023 [20,21]. In contrast to EuroMOMO’s approach of ignoring mortality between 2020 and 2022, CDC’s approach replaced mortality observed during outlier weeks with mortality expected in the absence of a pandemic [10]. To the best of our knowledge, no all-cause excess mortality estimates in the current post-acute pandemic period are publicly available for the US; excess mortality from pneumonia and influenza continue to be reported [22]. This replacement or exclusion of observed mortality, for part or whole of the acute pandemic years, has been used by public health agencies in other countries as well [23,24].

We believe that there are at least two interpretations of excess mortality going forward, including: mortality in excess of what would be expected if trends before emergence of the pandemic continued, and mortality in excess of what is expected with COVID-19 as an endemic disease. Support for the latter case arises because of the likelihood of SARS-CoV-2 remaining a circulating pathogen. While there remains substantial uncertainty in infection fatality rate and transmission dynamics of SARS-CoV-2 in non-naive populations, the shift of influenza A(H1N1)pdm09 from pandemicity to a key contributor to seasonal influenza burden is a likely precedent [25,26]. Consequently, we believe that the latter interpretation of excess mortality, one where COVID-19 (like influenza, respiratory syncytial virus (RSV) and other seasonal respiratory diseases) remains a contributor to mortality, with uncertainty in both the magnitude and timing of its mortality costs, to be more reasonable. Mortality with COVID-19 as a cause of death needs to be factored in when estimating mortality expected from all causes.

Given the heterogeneity in the pandemic’s impact across (and within) countries and age groups, approaches that rely on ad hoc specification of the start of the endemic phase of COVID-19 and/or manipulation of observational data should be avoided in favour of models robust to outlier weeks of high mortality during the pandemic. In this study, we describe two methods for national all-cause mortality monitoring and demonstrate their use with publicly available all-cause mortality datasets for Europe and the US. Central to interpreting estimated excess mortality is a clear understanding of the counterfactual modelled. Here, the outcome of interest is weekly number of excess deaths, irrespective of cause and the models aim to predict the number of deaths expected from all causes (henceforth, baseline), based on trends and seasonality seen in the past, without selectively excluding or substituting mortality during prespecified weeks/periods. 

## Methods

### Data sources

 For 19 European countries (Austria, Belgium, Bulgaria, Croatia, Estonia, Finland, Hungary, Iceland, Lithuania, Latvia, the Netherlands, Norway, Poland, Portugal, Slovakia, Slovenia, Spain, Sweden, Switzerland), national weekly deaths from all causes, disaggregated by sex and 20-year age groups (< 20 years, 20–39 years, 40–59 years, 60–79 years and ≥ 80 years) were retrieved from the Eurostat database [27]. Annual population estimates for these groups were also retrieved from Eurostat [28]. Data for 10 or more pre-pandemic years was available for these countries.

In the US, a shorter historical record of weekly mortality was available from public data sources. Weekly counts of deaths by state and age from 2018 onwards were extracted from CDC WONDER’s multiple cause of death interface [29]; data for 2015–17 were retrieved from a static dataset from the National Center for Health Statistics [30]. While both datasets allow stratification by 10-year age groups, they suppress instances with fewer than 10 deaths; consequently, we found modelling mortality in the very young (< 20 years), as well as including sex as an additional stratification alongside age would not be feasible with these sources. Use of larger age groups (< 45 years, 45–65 years, 65–74 years, 75–84 years, and ≥ 85 years) helped reduce suppressions in most states and our analysis was limited to these states (37/50, 74%). Results for Europe are reported in the following sections, and for US in the Supplement – United States.

### Methods overview

In this section, we describe two models for national all-cause mortality monitoring: (i) a generalised additive model (GAM) used by the WHO [3,31] to estimate excess mortality during 2020 and 2021, which we adapted to incorporate changes in population size, group-specific annual trend and within-year seasonality (both modelled as smoothing splines); and (ii) a generalised linear model (GLM), with a Serfling formulation of within-year seasonality in mortality [32] (like the EuroMOMO model) and breakpoints to detect changes in trend [33,34]. These models were designed to meet characteristics we believe to be desirable in mortality surveillance methods generally, as outlined in the Box, in addition to considerations specific to the post-acute phase of the pandemic as described in the previous section.

Box **Target features of models for mortality monitoring**

- Ability to model group-specific trends and seasonality in mortality when they are expected to differ between groups. As mortality has a clearer seasonality among the elderly population (> 65 years), and drivers of trends in population and mortality differ by age group [44,45], age is probably the most important grouping, but models must support inclusion of additional stratifications depending on operational needs;
- Support for reporting group-specific estimates to help detect occurrence of excess mortality in a subset of groups even when there is no excess in the aggregate;
- Ability to provide fuller representations of the distribution of expected mortality than point estimates (such as mean or median). Reporting prediction intervals and preferably prediction intervals at different confidence levels can better capture uncertainty and help aggregation across groups and/or time.; and
- Ability to estimate excess mortality at weekly frequency. To be included in public health surveillance reports, real-time approaches should model weekly seasonality, provide weekly estimates and address partial, i.e. delayed or incomplete, mortality reporting in recent weeks. This is in contrast to retrospective analyses where quarterly or annual estimates may be sufficient.

#### Generalised additive model (GAM)

This is an extension to a model used by the WHO to calculate monthly excess mortality during the first 2 years of the pandemic [3,31]. If *y_t_* is all-cause deaths during month *t* among the population in a state/country, a negative binomial model of expected deaths was specified as:


$$y_{t}|\mu_{t},\;\phi_{t}=NegBin\left(\mu_{t},\;\phi_{t}\right)$$

$$log\left(\mu_{t}\right)=\;f^{y}\left(year\left(t\right)\right)+\;f^{m}\left(month\left(t\right)\right)$$

where *μ_t_* and *ϕ_t_* are the mean and overdispersion parameters. The mean was modelled as a linear sum of an annual trend term (*f^y^*, modelled as a thin-plate spline) and a within-year seasonal term (*f^m^*, a cubic cyclic spline [35]).

Here, we retained this basic form and used weekly frequency instead of monthly and made the following changes: (i) addition of annual population as an offset to account for the change in population over the training period, (ii) estimation of separate annual trend and seasonal terms for each group, and (iii) addition of group as a fixed effect. Specifically, for the US, each age category had separate annual and seasonal terms, and for European countries, where data by decedent sex was also available, separate annual trend was modelled for age–sex combination. As there was no indication of different seasonality in mortality of men and women within an age group, this term was only age-specific.

If *P_tk_* is the population of group *k* at time *t*, the mean was modelled as:


$$log\left(\mu_{tk}\right)=\ln{(P}_{tk})+k+f_{k}^{y}\left(year\left(t\right)\right)+\;f_{k}^{w}\left(week\left(t\right)\right)$$

where *f^y^_k_* and *f^w^_k_* are the group-specific annual and weekly smoothing functions, respectively, *week(t)* ∈ {1, 2, ..., 53} denotes ISO week number [36] and *k* is a categorical variable.

The model was implemented using the *stan_gamm4* function in R package *rtsanarm* [37,38] using default weakly informative priors (Example R scripts are provided in the repository). Weekly estimates of expected mortality at different α-quantile levels were obtained from the posterior distributions of expected deaths, where α = {0.025, 0.05, 0.1, 0.2,…, 0.9, 0.95, 0.975, 0.25, 0.75} Estimates of weekly excess mortality were calculated from the distribution resulting from subtracting observed deaths from expected deaths in each sample.

#### Generalised linear model (GLM) with break points

This model combined features of a newly proposed model from the United Kingdom (UK)’s Office for National Statistics (UKONS) [24] and the EuroMOMO model [18]. Following UKONS specification, we trained a negative binomial GLM with mortality as outcome, population as an offset, separate terms for group, a trend component, as well as interaction terms between group and trend. To capture weekly seasonality, we included a seasonal term along the lines proposed by Serfling and in use by EuroMOMO [32]. Unlike UKONS model, terms for subnational regions were not included, i.e. groups were limited to age (in the US) or age–sex combinations (in Europe).

A more substantial update was in estimating trend. As implemented by UKONS, the trend is monotonic and linear, and when parts of the pandemic period were included in training the model, we found the model to identify an increasing trend for the entire training period, consequently overestimating mortality for 2–3 years leading up to the pandemic. To circumvent this issue, we used a seasonal trend decomposition using Loess (STL) [39] to identify trend in observed mortality, and an iterative heuristic-driven process to detect an optimal number and location of break points in the trend [33,34]. A full description of the approach and illustrative examples are provided in Supplementary Text S1. The last detected trend in the training period was assumed to continue during the projection period. Note that this procedure is not required to detect break points, i.e. the optimal number of break points can be 0. If *y_tk_* and *P_tk_* are as defined above,

$$log\left(\mu_{tk}\right)=\ln{(P}_{tk})+k+g_{k}(t)+\;h_{k}(t)$$

$$h_{k}\left(t\right)=sin\left(\frac{2\pi t}{52.14}\right)+cos\left(\frac{2\pi t}{52.14}\right)$$

where *h_k_(t)* is the cyclic function for weekly seasonality following Serfling specification, and *g_k_(t)* is a segmented regression function for trend. The models were implemented using the *stan_glm.nb* function in *rtsanarm* package [37]; estimation of quantiles for expected and excess deaths are as described for the GAM.

### Estimating uncertainty

Uncertainty quantification in expected and excess deaths at different confidence levels as well as aggregation across groups and weeks was identical in both methods. For example, the 95% prediction interval (PI) of expected and excess deaths was calculated from 0.025 to 0.975 quantiles of the corresponding posteriors; 0.5 quantile was the point estimate. The use of multiple quantile levels allows a more flexible assessment of uncertainty, and estimates at other confidence levels can be calculated by picking the appropriate quantiles — 0.05 and 0.95 for 90% PI, 0.1 and 0.9 for 80% PI, etc.

National mortality was not modelled separately but was an aggregation of the posteriors across all age (and sex) groups. Estimates for any subset of groups (e.g. among women across all ages, among those younger than 65 years) was obtained by a similar aggregation of appropriate posteriors. Estimates for a month, quarter or any arbitrary period can be aggregated from the posteriors of the corresponding *n*-week window.

The fraction of samples predicting an excess during a week was used as a probability of excess deaths in the week. This quantification of confidence on a continuous scale is an alternative to dichotomous reporting of statistically significant excess mortality. In other words, to the conventional excess mortality question — How many excess deaths were there in a given week? — a supplementary question was added: What is the probability that there were *any* excess deaths in a week [5]?

### Generating projections

In Europe and the US, national all-cause mortality tends to have a clear seasonality with a trough in summer months and a peak during winter. We assumed that, operationally, estimates of expected mortality would be generated once per year, around week 40, in preparation for the winter season. Models were fit using weekly mortality up to June (specifically, up to and including ISO week 26, denoted as W26) and estimates generated for the next 52 weeks (W26 of the next calendar year). This design ensures that, at the time projections are made, all observational data used to fit models are complete, and retaining projections unchanged during the year obviates the need to correct for data incompleteness within the model. Limiting the projection period to one year also renders the GLM method’s assumption of a continuation of the last detected trend into the entire projection period more reasonable. Estimates of excess mortality (observed − expected), on the other hand, are updated weekly as new observational data are reported and hence sensitive to mortality anomalies and completeness of mortality reporting. Note that this is different from settings, more common in outbreak detection, where models are retrained with recent observational data.

We projected mortality expected under the following scenarios: (i) models fit to mortality seen through week 26/2023; (ii) models fit to mortality seen before the pandemic, i.e. up to week 52/2019; and (iii) models fit to mortality data seen through week 26/2022. In all cases, projections were made until week 26/2024. The first setting is the closest to the hypothesised operational use in surveillance for the 2023/24 season, and the latter two demonstrate the effect of inclusion of mortality observed during the acute pandemic period on models’ fit and expected mortality. For the European dataset, historical data from week 1/2010 up to the aforementioned cutoffs were used, and for the US, data from week 1/2015 onwards were used. For demonstrating model fit in the following section, we consistently use Finland as an example and show plots for other locations in Supplementary Figures S3 and S4.

### Validation metrics

In addition to generating projections in the above hypothesised operational setting, we generated retrospective projections in the pre-pandemic period. For each of the years 2010 to 2018, we fit models to mortality during the previous 10 years (moving window) and projected weekly mortality for the next 52 weeks, identical to the above hypothesised operational setting. In other words, we verified whether the models could have predicted weekly mortality in the years leading up to the pandemic if they had been in use in this period. This is an essential check of out-of-sample skill and calibration of the models. Metrics used for validation are described in Supplementary Text S2. We evaluated the models’ estimates across all countries represented in the Eurostat dataset; a similar evaluation was not possible with the US dataset given its shorter historical record.

## Results

**Figure 1:**
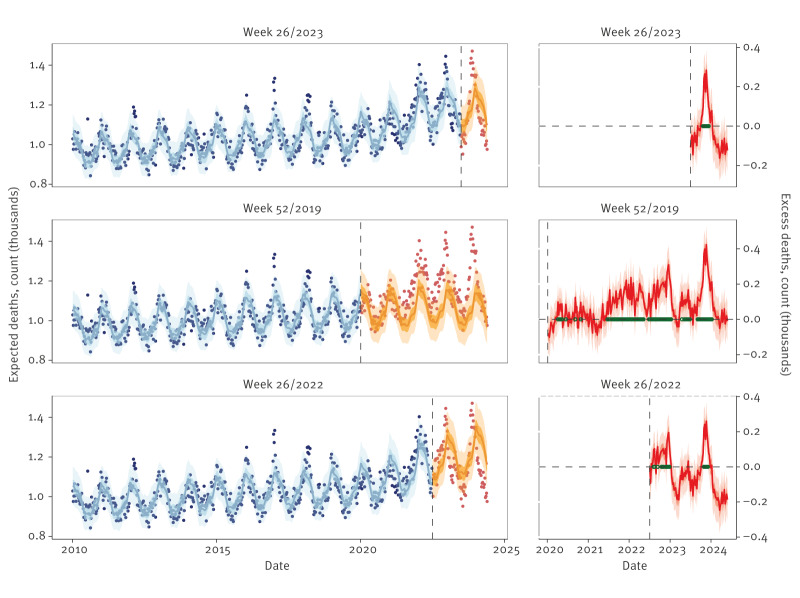
Estimates of expected and excess deaths from a generalised additive model fit with mortality observed through three different endpoints, Finland, week 1/2020–week 26/2024

**Figure 2:**
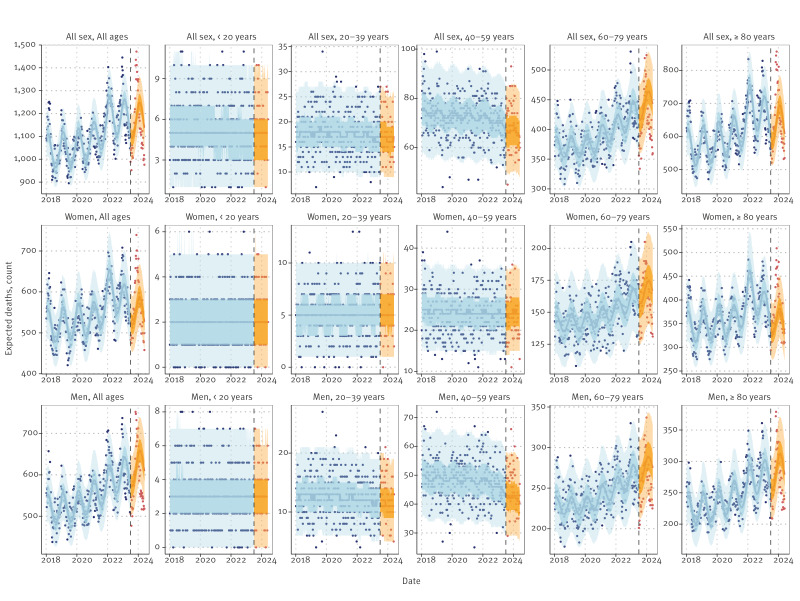
Age- and sex-stratified estimates of expected and excess deaths from a generalised additive model fit with mortality observed through week 26/2023, Finland, week 1/2018–week 26/2024

Visual inspection of estimates indicated that the models could capture pre-pandemic trends and seasonality when trained on data through 2019 (Finland, Figure 1 with GAM and Supplementary Figure S1 with GLM). With inclusion of data up to week 26/2022, an increasing trend during the pandemic years was detected, which stabilised with the inclusion of mortality observed between week 27/2022 and week 26/2023. Group-specific fits (Figure 2 and Supplementary Figure S2, for GAM and GLM, respectively) demonstrated the models’ ability to discern patterns among age and sex groups. For example, in Finland, no change in the mortality trend was detected in the younger age groups (< 20, 20–39 and 40–59 years) with the inclusion of mortality over 3 years of the pandemic period. Supplementary Figures S3 and Figure S4 show corresponding estimates for other modelled countries in Europe and the states in the US, respectively.

In-sample fit of the generalised additive model (blue), expected deaths (orange), and excess deaths (red, right panels) using mortality observed through week 26/2023 (top row) week 52/2019 (middle row), and week 26/2022. The centre band (dark shading) shows interquartile range (0.25–0.75 quantiles), and outer band (light shading) shows 95% prediction interval. Data points show reported mortality. Weeks with statistically significant excess are indicated by a green point on *y* = 0 line (right).

Each subpanel shows model fit (blue) and expected weekly mortality (orange) between week 27/2023 and week 26/2024 for one age–sex group. Aggregated estimates across age and sex are shown in the first column and first row respectively; overall national estimates are shown in the top left panel. Centre darker band shows interquartile range (0.25–0.75 quantiles), and outer band shows 95% prediction interval. Data points show reported mortality; only observations available through week 26/2023 (blue) were used to train the model.

**Figure 3:**
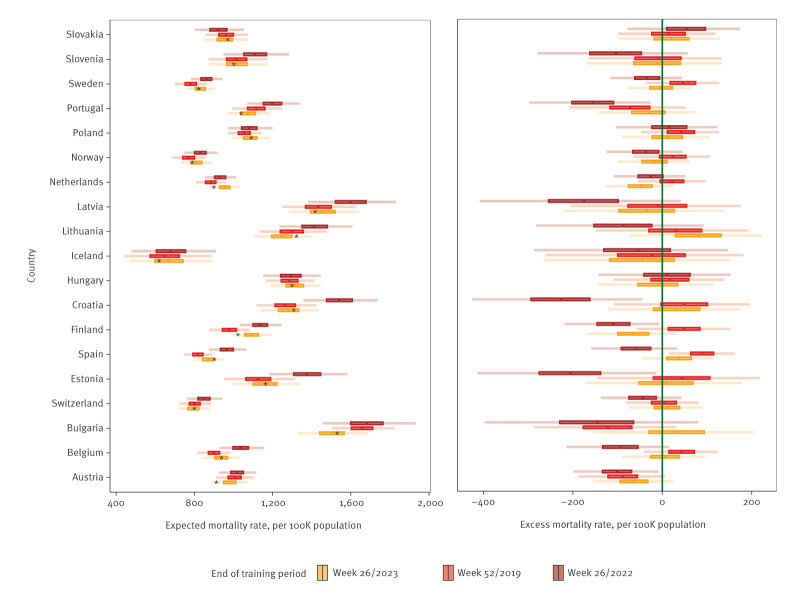
Annual estimates of expected and excess mortality per 100,000 population from generalised additive models trained on mortality observed through three different endpoints, 19 European countries

**Figure 4:**
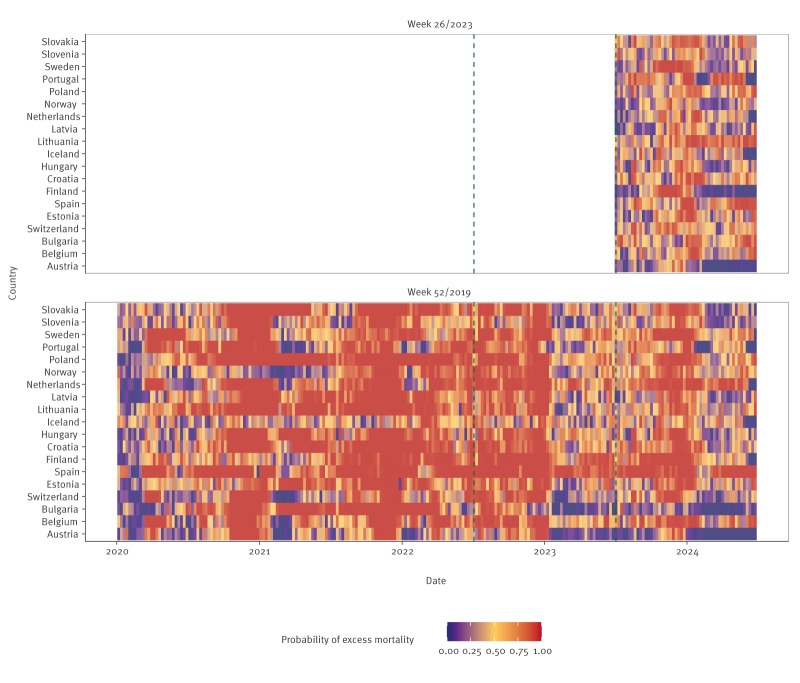
Probability of excess mortality during one week, as estimated by the generalised additive model, using mortality observed through week 52/2019 and week 26/2023, 19 European countries

On an annual scale, the effect of inclusion of mortality observed in the acute pandemic period on expected and excess mortality for the most recent surveillance year between week 27/2023 and week 26/2024, was primarily an increase in uncertainty (Figure 3). This widening of the prediction intervals is clearer when only estimates from the early pandemic period were included (up to week 26/2022). Figure 4 indicates that, in almost all countries, there were weeks where estimated excess mortality was likely, even when no excess mortality was estimated for the year overall.

Model estimates of expected mortality rate when trained on mortality observed through week 26/2023 (orange), week 52/2019 (red), and week 26/2022 (brown) are shown in the left panel, and corresponding estimates of excess mortality are shown in the right panel. Star (⋆) denotes observed mortality rate for the period. The lighter and darker bands show 95% and 50% prediction intervals, respectively. A deficit (negative excess) seen in a few countries when the model was trained on mortality observed through week 26/2022 (brown) may be interpreted as fewer deaths during the projection period than was expected from the change seen during the first 2.5 years of the pandemic.

The top panel shows the probability that there were *any* excess deaths in a country (y-axis) during a given week (x-axis) between week 27/2023 and week 26/2024 based on mortality observed through week 26/2023. Weeks where the models were confident of an excess are shown in dark red. The lower panel shows the probability of excess mortality when only mortality observed before the start of pandemic, i.e. week 52/2019, was used to train the model.

**Figure 5:**
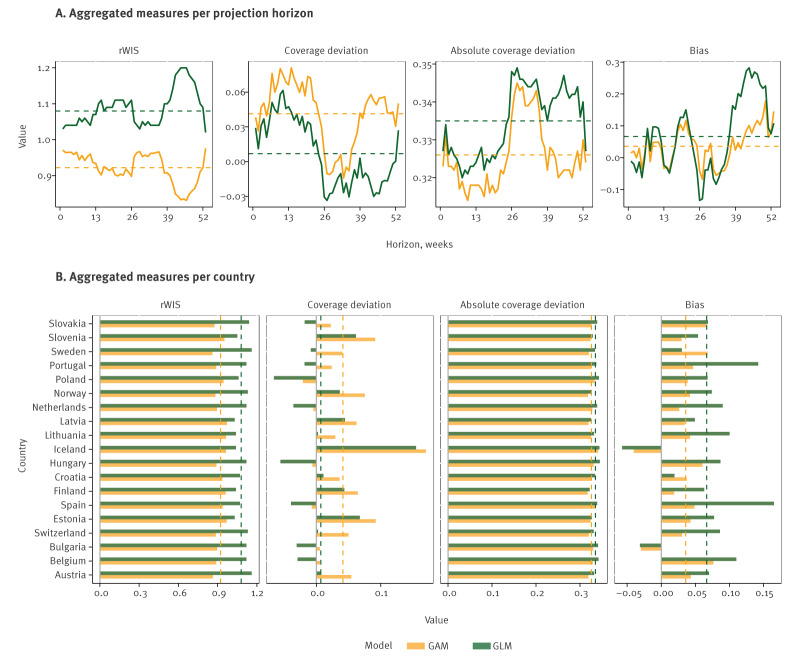
Calibration measures for GAM/GLM models, summarised across 19 countries, years, age–sex groups and projection horizons, 2011–2019

GAM: generalised additive model; GLM: generalised linear model; rWIS: relative weighted interval score.

Measures from cross-validation indicate that both models were positively biased and had lower coverage at the turn of the calendar year, coinciding with the period of usually high respiratory disease activity in the region (Figure 5). Of the two models, GAM had better overall skill (lower relative weighted interval score, as defined in Supplementary Text S2), calibration (absolute coverage deviation) and less variability in bias across countries. Both models had good interval coverage at different uncertainty level, with indication of a lower coverage at longer projection horizons for the GLM model (Supplementary Figure S5).

Measures are defined in Supplementary Text S2. Top panel shows measures aggregated across all countries, age–sex groups and years, for projection horizons of 1 week to 52 weeks (x-axis). The dashed horizontal line represents further aggregation across all weeks. Lower panel shows aggregated measures for each country with the vertical dashed lines denoting average across all countries (identical to the horizontal lines in the top panel). Absolute smaller values indicate better calibration.

Checks of posterior predictive distributions of both models suggest difficulty simulating low mortality only in the very young (< 20 years) as shown in Supplementary Figures S6 and S7. Inspecting model coefficients in each country was beyond the scope of this analysis, and broadly: (i) GAM often estimated weekly seasonality only in the older groups (60–79 years, ≥ 80  years), (ii) annual trend estimates were responsive to mortality during the pandemic period (as shown in Supplementary Figure S8), (iii) age was estimated to have the largest effect among terms included in the GLM, and (iv) interaction between age and annual trend had a stronger association than interaction of age and seasonality (as indicated by the model estimates reported in Supplementary Figure S9).

**Figure 6:**
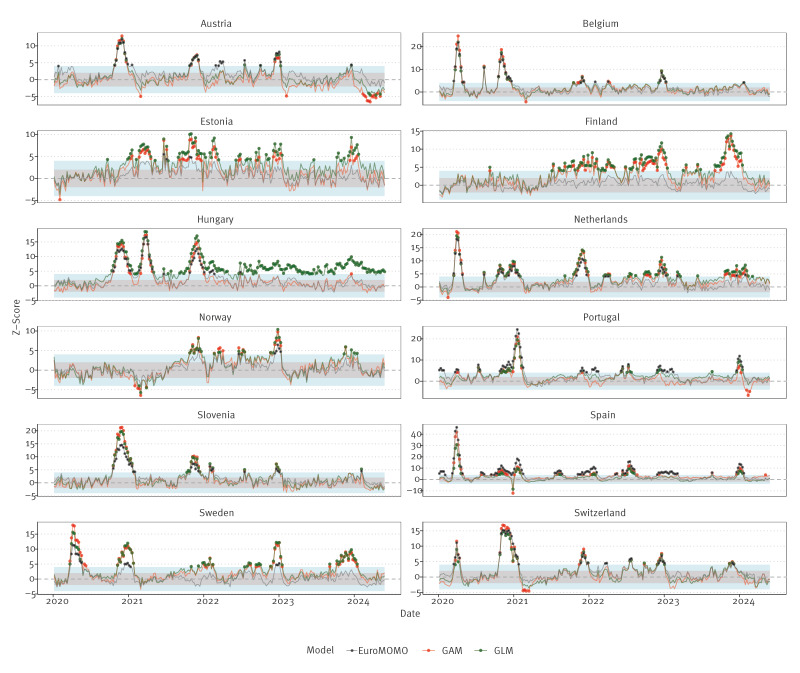
Comparison of z-scores estimated by EuroMOMO and GAM/GLM models, 12 European countries, week 1/2020–week 26/2024

EuroMOMO: European Mortality Monitoring network; GAM: generalised additive model; GLM: generalised linear model.

We also undertook a comparison of excess mortality estimates from models described here with those published by EuroMOMO. When training its model, EuroMOMO excludes mortality observed during winter months when a majority of deaths from influenza, pneumonia and related respiratory infections occur in Europe. As GAM/GLM did not exclude these months, the outcome being modelled here is not identical to that of EuroMOMO, and consequently, definitions of ‘excess’ also differ. For weeks 1/2020 through week 26/2024, GAM/GLM were found to flag a deviation for expected mortality more often than EuroMOMO, with variability in the frequency of deviation across countries (Figure 6). Supplementary Text S4 has a more detailed discussion. 

Estimates of z-scores were generated using mortality observed through week 52/2019 from the EuroMOMO model (black), GAM (red) and GLM (green). Following EuroMOMO’s convention, a ‘normal’ range was defined as −2 < z-score < 2, (shown in grey) and an ‘increase’ if 2 < z-score < 4 (in blue). Data points are plotted only when they fall outside the shaded regions and indicate a ‘substantial’ change.

## Discussion

Our results show that models that can adapt to mortality anomalies seen during the acute COVID-19 pandemic period without a need for adjustments to observational data or tailoring specifications for location or demographic group are feasible. Our methods were shown to provide reliable and well-calibrated distributional estimates and can complement approaches for mortality surveillance currently in use at collaborative networks like the EuroMOMO and national public health agencies. The resulting effect estimates are interpretable. Refinements and improvements within the proposed framework can be further explored, contingent on data availability and operational requirements.

Of the two models presented, there is no strong evidence from the experimental results to prefer either model for operational use, with possibly quicker adaptation of GAM method to changes in mortality (Supplementary Text S3). It has also not been possible to evaluate their calibration against other mortality models because of limited public availability of a historical record of their distributional estimates. Model combinations (or model averaging), which are shown to have improved skill in multiple domains including infectious disease surveillance when no clear superior model can be identified beforehand, could be pursued in real-time mortality surveillance as well [40,41].

As stated earlier, excess mortality relative both to pre-pandemic levels and to mortality shifts seen during the COVID-19 pandemic can have operational value in routine surveillance. It is perhaps preferable to monitor excess mortality under both scenarios for the next 2–3 years, until there is increased clarity on the interaction of mortality because of COVID-19 vis-à-vis that caused by other respiratory pathogens. We believe this recognition of COVID-19 endemicity in mortality surveillance is an essential and eventual step. It is also important to avoid modelling choices that inhibit a clear interpretation of the baseline, and hence excess, mortality. Relatedly, while a negative excess (or deficit) was estimated in a few countries when models were trained on mortality observed during the acute pandemic years, the models were able to subsequently flatten this trend.

Our analysis has a few limitations, primarily, with insufficient handling of change in age composition, of lags in mortality reporting that can delay detection of excess mortality and of cause attribution. Changes in population size and age composition over time should be accounted for when predicting national all-cause mortality. In the models described above, the use of population as an offset adjusted for change in population size. Use of age-specific parameters can account for change in age composition when the age categories are not too broad, but because of constraints imposed by publicly available datasets, the broad age categories used here may have been insufficient to capture change in composition nationally, and within each category as well. A related artefact was an inability to model mortality in a quarter of the states in the US for which mortality data were suppressed. Sensitivity of model calibration to the length of the baseline period has also not been verified. Better resolved data can help complete some of these checks which are essential before operationalising these methods.

The second limitation stems from the several week lag/delay in the reporting of final weekly mortality. While this incomplete reporting would not impact estimates of expected deaths, because the models described are not retrained every week, they would delay detection of deviations from expected, i.e., excess, mortality. Correcting for these issues is essential for real-time mortality surveillance and can be seen as a distinct and separate concern from estimating expected mortality. Delay correction methods have been explored in other surveillance contexts [42,43], and adaptation of these approaches for all-cause mortality in Europe would greatly benefit from availability of publicly accessible versioned datasets of delays in mortality reporting. 

Lastly, cause-specific mortality monitoring is a vital part of surveillance alongside all-cause mortality, and the suitability of the models proposed here for cause-specific mortality and specifically causes with more complex trends or seasonality remains to be evaluated. Influenza and other respiratory diseases, a commonly used category in cause-of death reporting, is among the top 10 leading causes of death in most European countries and the US, and endemic COVID-19 can potentially have a substitution effect on mortality from these causes. It is important to note that excess mortality identified by the above models trained on all-cause mortality cannot be attributed to a specific cause. Similarly, caution is required in comparing these models with those that are trained to predict mortality from a subset of causes (Supplementary Text S4 provides a more detailed discussion).

## Conclusions

While the larger effects of the COVID-19 pandemic on all-cause mortality are possibly behind us, these past periods of anomalous mortality remain a challenge for routine mortality surveillance. We believe that surveillance methods that adapt to these periods are feasible, can provide reliable and calibrated estimates, and may be of value alongside methods currently in use at national public health agencies and inter-agency networks.

## Data Availability

Analysis scripts, input data and sample model outputs are available at: https://github.com/fhi-kan/excess_mort_surv

## Supplementary Data

4176000 Supplement

## Supplementary Data

1349000 Supplement_United_States
