# Supplementary material for: Real-time monitoring of excess mortality under a new endemic regime
Source: Euro Surveill. 2025 Jun 26;30(25):2400753. doi: 10.2807/1560-7917.ES.2025.30.25.2400753 (PMC12207195; doi:10.2807/1560-7917.ES.2025.30.25.2400753)
Supplement: Supplement [file 24-00753_KANDULA_Supplement_MAIN.pdf]

This supplementary material is hosted by Eurosurveillance as supporting information alongside the article Real-time monitoring of excess mortality under a new endemic regime *on behalf of the authors who remain responsible for the accuracy and appropriateness of the content. The same standards for ethics, copyright, attributions and permissions as for the article apply. Eurosurveillance is not responsible for the maintenance of any links or email addresses provided therein.*

Contents

1. Supplementary Text S1: Breakpoint detection for the GLM model
    - supporting figures *TextS1-F1* and *TextS1-F2*
2. Supplementary Text S2: Temporal cross validation
3. Supplementary Text S3. Extended validation
    - supporting figures *TextS3-F1*
4. Supplementary Text S4. Comparison with EuroMOMO estimates
    - supporting figure *TextS4-F1*
5. Supplementary Text S5. Prospective projections for 2024-2025
    - supporting figures *TextS5-F1* and *TextS5-F2*
6. Supplementary Figures S1-S9

Supplementary Text S1: Breakpoint detection for the GLM model

Seasonal and Trend decomposition using Loess (STL) was used to decompose observed mortality to a weekly seasonal and trend component (see TextS1-F1 below for example)(1, 2). As Serfling formulation (3) is often used for modeling seasonality of all-cause mortality, the corresponding component from STL decomposition was ignored and we focused on the trend component. We hypothesized that the effect of including the mortality seen during the acute pandemic years in the model fit, would be an upward shift in the mortality trend. To avoid an erroneous identification of an increasing trend over part of the pre-pandemic period due to pandemic mortality, and of manually imposing an increase starting with the emergence of SARS-CoV-2, further complicated by group specific differences in mortality, we adopted a heuristic-driven segmented approach. Intuitively, we tried to model a change in trend due to the pandemic, but the start (i.e. break point) was detected algorithmically for the different locations, age and sex groups. This approach has the additional advantage of possible detection of a levelling of trend anticipated during the endemic phase of Covid-19.

Segmented regression methods model the relation between response (trend component of STL) and one or more explanatory variables as two or more straight lines connected at unknown values (break points). We used the *selgmented* function in R's *segmented* package for detecting an optimal number of break points as well as to fit a regression line for each segment. Readers are referred to relevant articles and package documentation for details (4-6); R scripts appended to this manuscript has implementation.

To summarize, the selection of the number of breakpoints relies on a sequential hypothesis testing procedure (6). It starts with testing a null hypothesis of no break points (H0) against an alternative hypothesis of *Kmax* breakpoints (H1). If H0 is rejected, the number of breaks points under H0 is increased by 1, and conversely, if H1 is rejected, breakpoints under H1 are decreased by 1, and the process continues. *Kmax* was set to 2 (package default). The p-score test (5) was used to compare hypotheses but alternative information criterion measures, such as Bayesian Information Criterion, are also possible. Overall, the GLM model estimates did not appear to be sensitive to the maximum number of break points (*Kmax*). For continued use of the model in subsequent years, *Kmax* could be increased to better capture changes.

An illustrative example below (TextS2-F2) shows change in location of break points when mortality during 2020-2023 was included. Break points were detected in early 2020 for 60-79 year and 80+ year groups only.

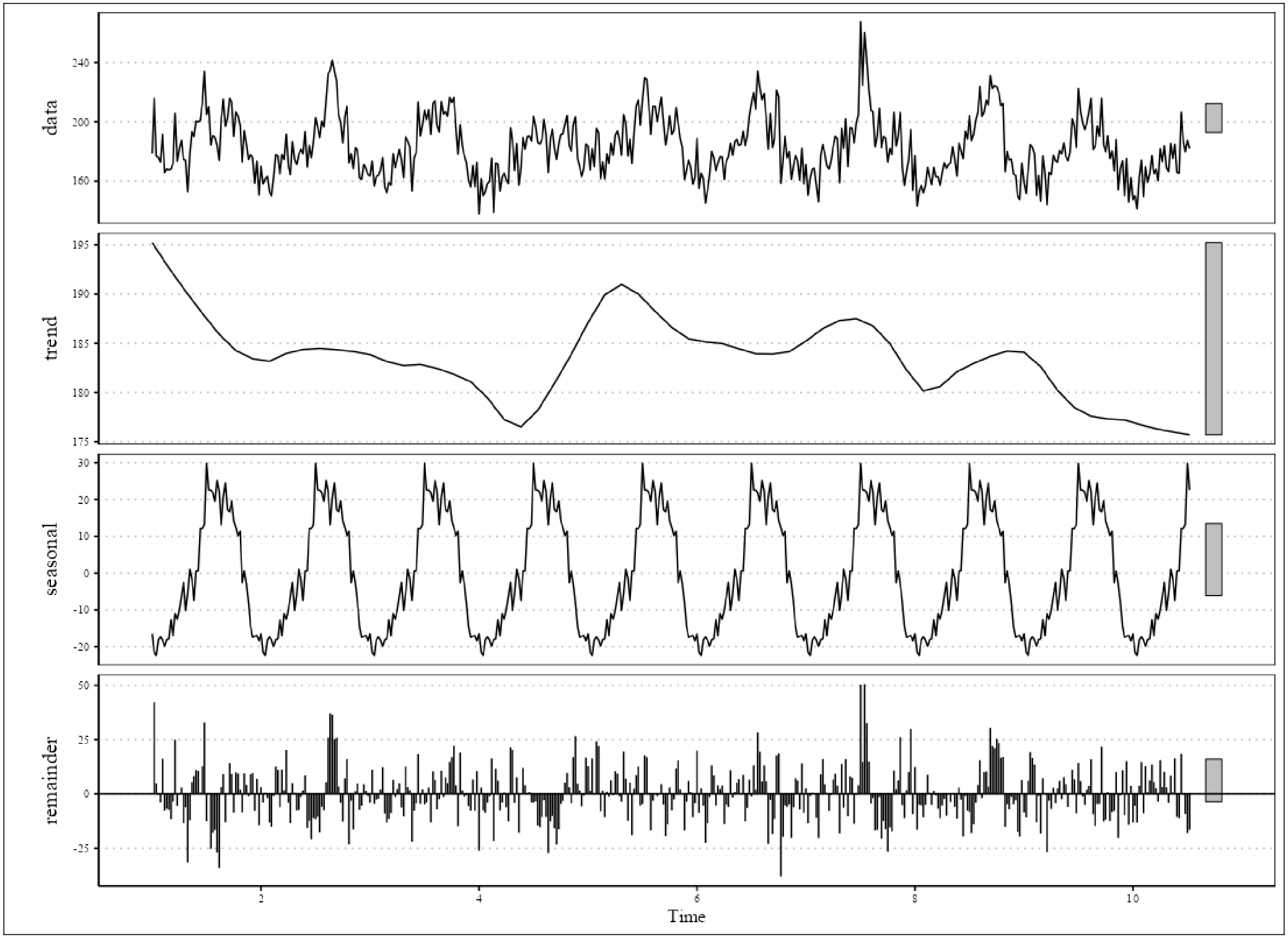

TextS1-F1. Weekly mortality in Finland (top) between 2010-W27 and 2019-W52, and its components -- trend, seasonality, remainder – detected by STL. The bar on the right of each panel represents the same length and can be used to compare scale of the different components.

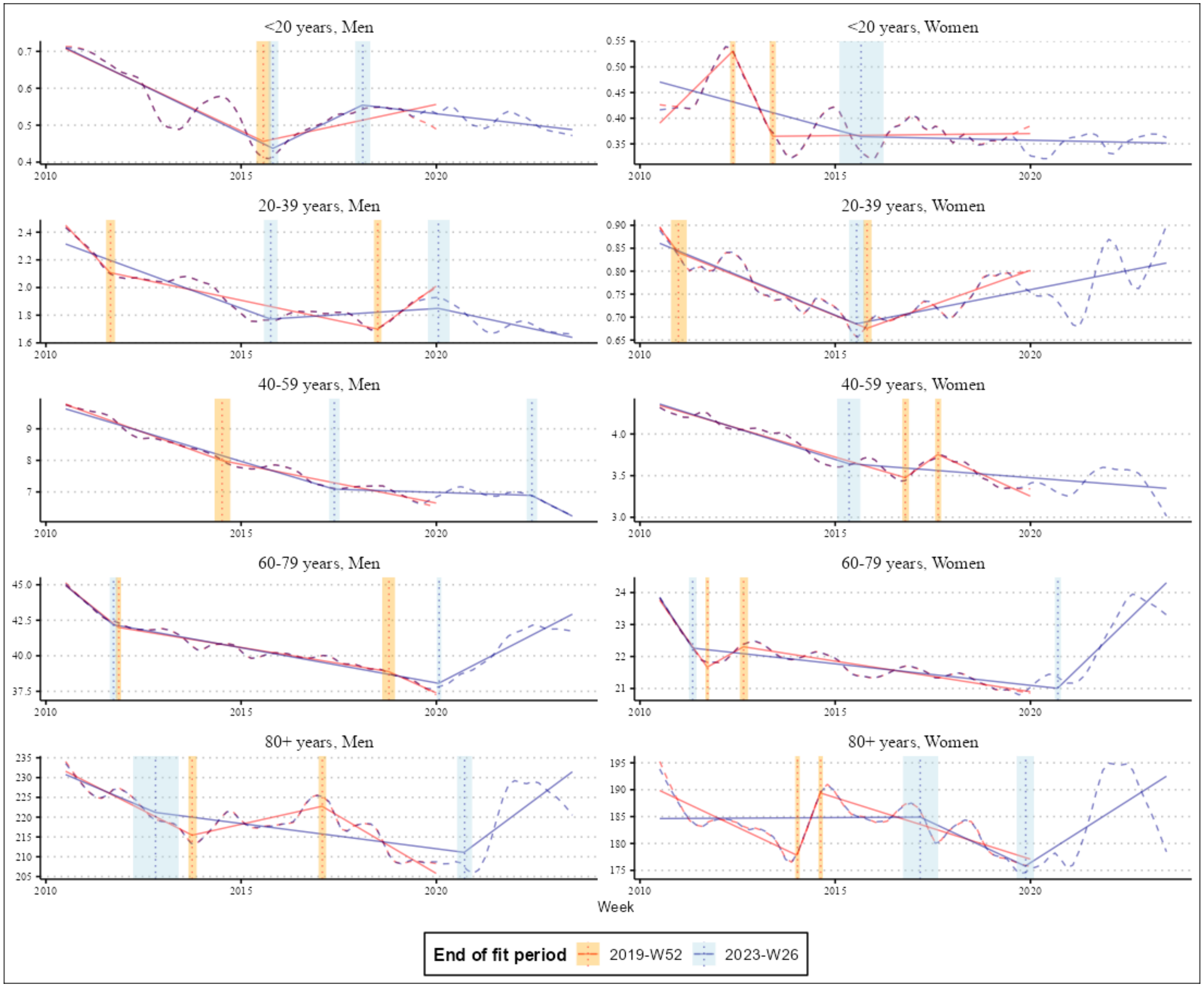

TextS1-F2. Observed mortality by age and sex groups in Finland. The dashed blue line shows mortality, and the solid lines represent the estimated slope for each segment using mortality through 2019-W52 (red) and 2023-W26 (blue). The colored vertical regions show break points and confidence interval (10%-90%). Note different range of y-axis in each panel.

Supplementary Text S2: Temporal cross validation

To inspect model estimates of expected deaths during the years before the pandemic, for each of the years 2010-2018, we trained the models with 10-year data up to W26 of the candidate year and projected mortality one year ahead. From the quantile distribution of the forecasted mortality rates, we calculated three measures of forecast skill and calibration, as implemented by Bosse et al (7).

Interval score: The interval score is a proper scoring measure for quantile forecasts (8, 9). For a single quantile level α, it can be interpreted as the sum of penalties for dispersion (the width of the interval), overprediction (observed value below lower bound of the interval) and underprediction. Score for a probabilistic forecast expressed at *k* levels was calculated as a weighted sum of the interval scores (WIS) of the different levels. When estimates across multiple locations and period needed to be summarized, i.e. observations of unequal range, the model's skill relative to a 'hypothetical average model' was calculated (rWIS) (10).

Coverage: *Interval* coverage was calculated as the proportion of observations that fall within a given prediction interval. A similar measure, *quantile* coverage, was calculated as the proportion of observed values less than the estimate for a given quantile level. For example, the quantile coverage at the 0.95 level is the proportion of observations that are less than the 0.95 quantile estimate, whereas the 95% interval coverage is the proportion of observations that are between the 0.025 quantile estimate and the 0.975 quantile estimate. *Coverage deviation* is a mean of interval coverages at the different quantile levels and can potentially cancel out oppositely signed deviations. To address this, we also calculated an absolute version of coverage deviation.

Bias: When the observed value was smaller (greater) than the median of the predictive distribution, bias was the maximum (minimum) percentile rank for which the prediction is smaller (greater) than the observation. Bias is zero if the observed value equals the median. It has a well-defined range of -1 and 1 and considered to be a robust indicator.

Aggregate summary measures reported are a simple average across all cross-validation years, locations, groups and horizons.

Supplementary Text S3. Extended validation

The overall objective of the cross-validation exercise included in the main text was to provide a measure of the models' predictive skill when used prospectively from 2024 onwards. Given that we do not have long historical periods when Covid-19 was endemic in the population, we do not have a clear comparable period. The assumption used in the main text, that the models' predictive skill in the pre-pandemic period, which did not have large mortality swings, can serve as a proxy for model skill in the Covid-19 endemic period, may not be entirely reasonable. We here show the effect of including the pandemic years in the validation period. Specifically, while in the main validation analysis, we fit models to mortality during the previous 10 years for each of the years 2010 to 2018 and projected mortality for the next 52 weeks, here we extend this 1-year out-of-sample projections by 5 more years, i.e. with training periods ending in each of 2019 to 2023.

This extension assesses the skill of a model trained primarily (or entirely) on pre-pandemic mortality in predicting mortality from all causes including Covid-19. A worsening of predictive skill is to be expected, but the magnitude of change can be informative. Neither alternative of validation periods (pre-pandemic or through 2023) may be truly reflective of model performance in the post-acute pandemic period.

TextS3-F1 shows a deviation of interval coverage beginning from autumn of 2020. As expected, due to large Covid-19 mortality during the different acute infection waves, fewer observations were within the expected intervals (colored lines below horizontal grey line). The deviation was smaller for the GAM model and returned to pre-pandemic levels sooner.

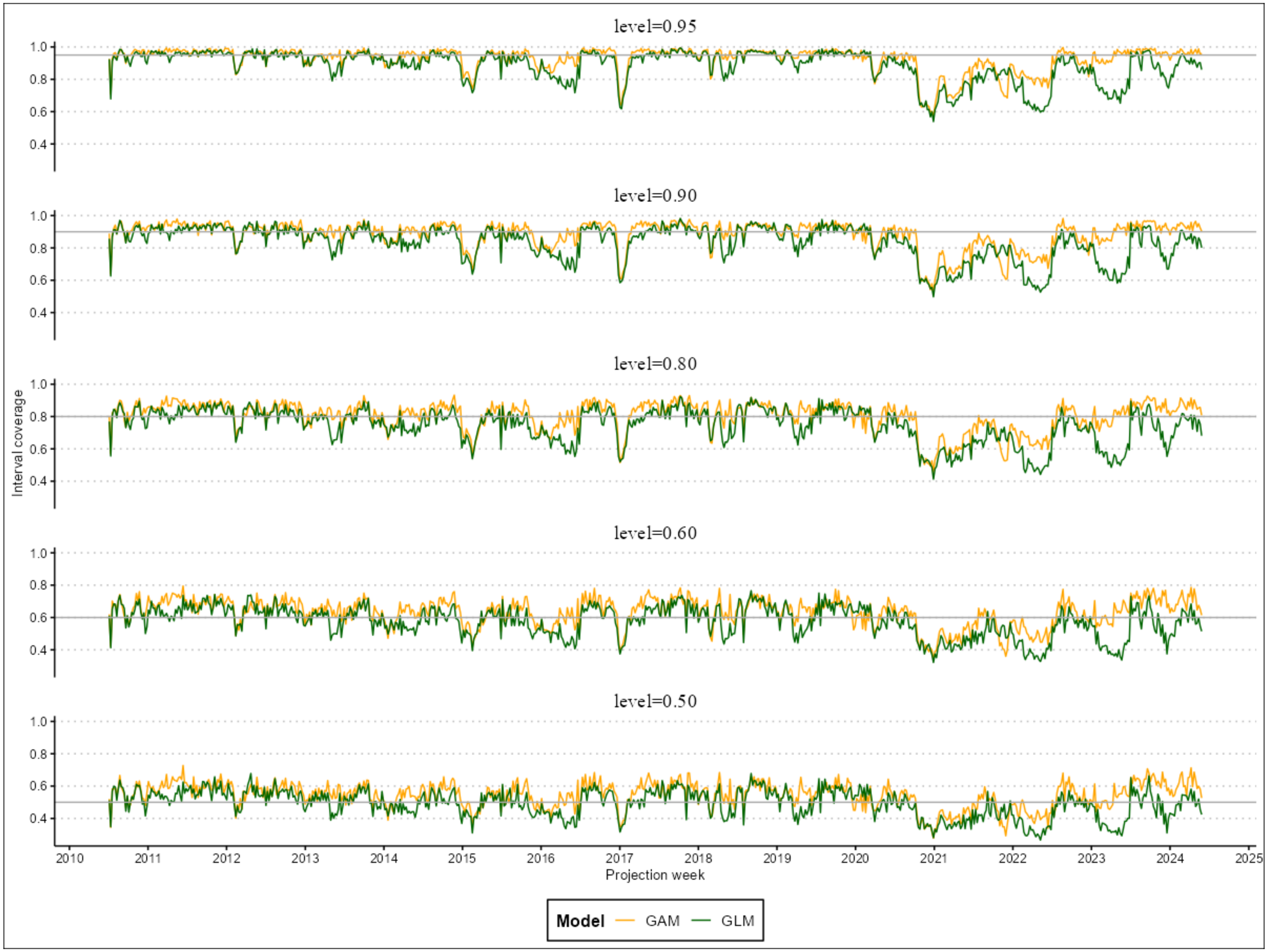

TextS3-F1. Interval coverage of the GAM (yellow) and GLM (green) for each week (x-axis), and at different confidence levels (sub-panels). The horizontal line in each sub-panel indicates expected coverage.

Supplementary Text S4. Comparison with EuroMOMO estimates

The European mortality monitoring initiative is a collaborative network for real-time standardized mortality monitoring across Europe. Part of the network's outputs include estimates of weekly excess mortality covering 26 countries in Europe hosted on a public website. While estimates of excess deaths are reported for all participating countries together, for individual countries, only z-scores are reported. These are standardized measures for comparing mortality across populations and periods, and are 'approximated as (number of deaths - baseline) / standard deviation of the residuals on the part of the series used to fit the model'(11). Details on their methodology and supporting publications are available (12).

Of note, these models are trained on mortality observed during select 22 weeks of a year (weeks 15- 26; 36-45), so that "only the period of the year when it is assumed that additional processes leading to excess deaths are not likely to happen [...]" are included (13, 14). Therefore, it is our understanding that the outcome modeled excludes, by design, mortality from pneumonia, influenza and other respiratory infections that predominantly occur during winter months in Europe. In contrast, the outcome of interest in this manuscript is all mortality irrespective of cause. This crucial difference has to be recognized when comparing estimates.

We downloaded weekly country-specific z-scores available on EuroMOMO website as of February 12, 2025, spanning weeks 2020-W01 to 2024-W26 (15). While EuroMOMO publishes z-scores at the national level as well as stratified by age group, the age groups differed from those used in this manuscript that were based on Eurostat data; hence, we limited comparison to the national level. To the estimates provided by the GAM and GLM models fit to data through 2019-W52, i.e. pre-pandemic period, we applied the above formulation of z-score. We also adopted EuroMOMO's practice of indicating a z-score between -2 and 2 as 'normal', and above 4 as 'substantial increase'. Of the 19 countries for which we used Eurostat data, 12 were covered by EuroMOMO. The following differences are apparent (Figure 6 in main text):

- the agreement between the three models varied by country and period;
- GAM/GLM indicated a substantial increase more often during the pandemic (i.e. z-score > 4);
- during weeks when models concurrently detected a substantial increase, the z-scores of the GAM/GLM models were likely to be larger in magnitude.

As stated above, due to the varying target definitions, the z-scores may not be directly comparable and these differences are to be expected. Including mortality observed during the acute phase of the Covid-19 pandemic through 2023-W26 and projecting deaths for the next year, showed better agreement among the models (TextS4-F1). The more noticeable deviations occurred in Portugal and Spain where EuroMOMO detected multi-week 'substantial increase' while GAM/GLM detected lesser (or no) excess; the deviation was in the opposite direction for Finland. Note, however, that EuroMOMO did not detect an excess in Finland during any of the weeks since 2020 (see TextS4-F1).

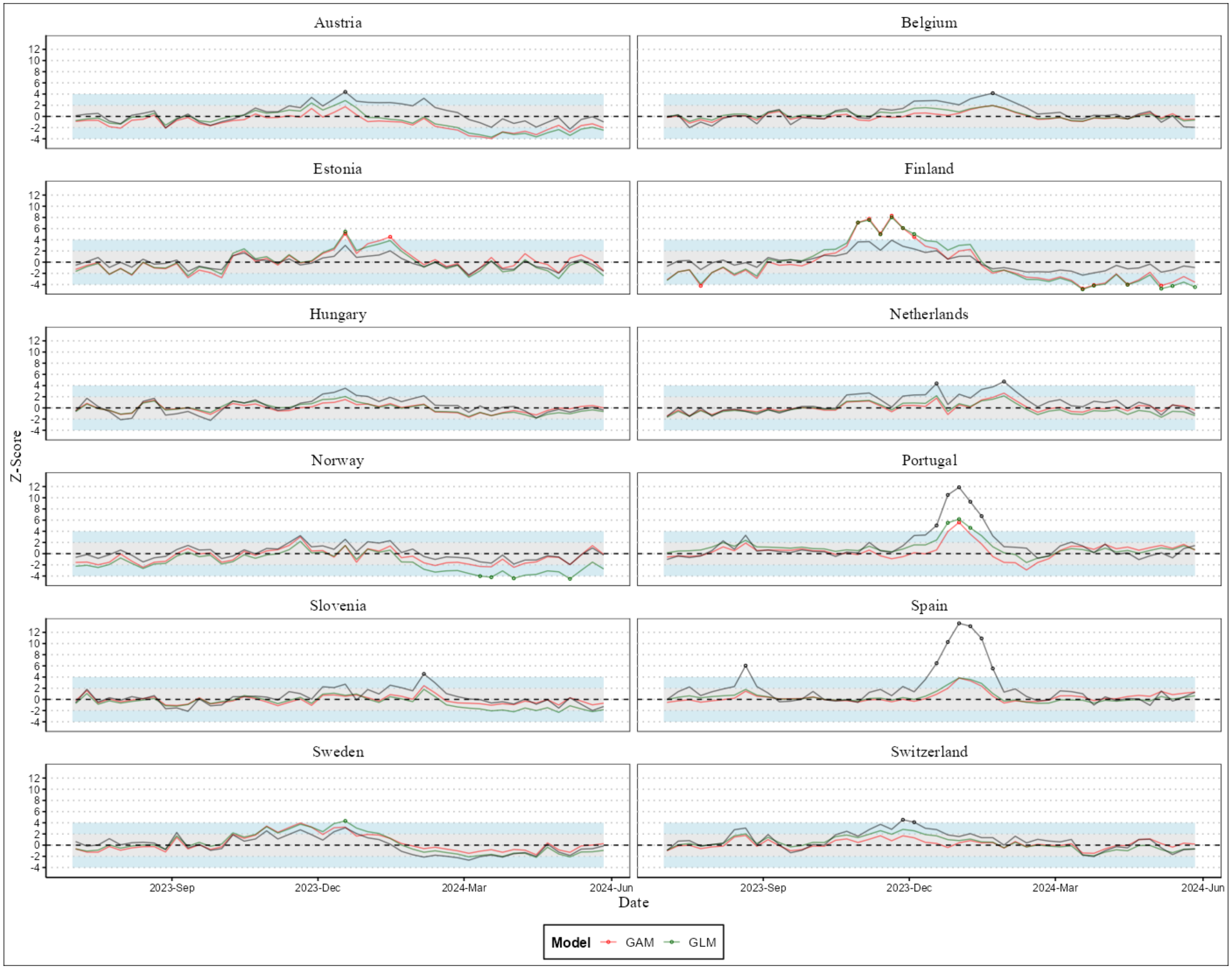

TextS4-F1. Estimated z-scores from the EuroMOMO model (black), GAM (red) and GLM (green) using mortality observed until *2023-W26*. Grey region denotes ‘normal’ range (-2<z-score <2); data points are plotted only when they fall outside the blue region (z-score>4 or z-score < -4), indicating a ‘substantial increase’.

Supplementary Text S5. Prospective projections for 2024-2025

The results presented in the main text were generated in the summer of 2024, and mortality data for some of the recent weeks at the time were either unavailable or provisional, limiting our ability to generate projections for the period between 2024-W27 and 2025-W26. Later revisions to the manuscript made in February 2025, were able to utilize these previously unavailable data.

In the figure below (TextS5-F1), we compare 95% prediction intervals of weekly expected mortality between 2023-W27 and 2025-W26, generated using mortality observed through 2023-W26 and data through 2024-W26 of the GAM model. The inclusion of an additional year's data has either retained the projections largely unchanged (Austria, Iceland, Latvia, Netherlands, Norway, Sweden, Slovenia) or resulted in an increase in level. The latter behavior was observed in countries where a greater mortality than expected was reported the previous year (for example, Lithuania), allowing the model to update its annual trend component.

Corresponding downward shift was seen in excess mortality estimates (TextS5-F2). Mortality reported for weeks in 2025 indicated significant under-reporting and hence not plotted.

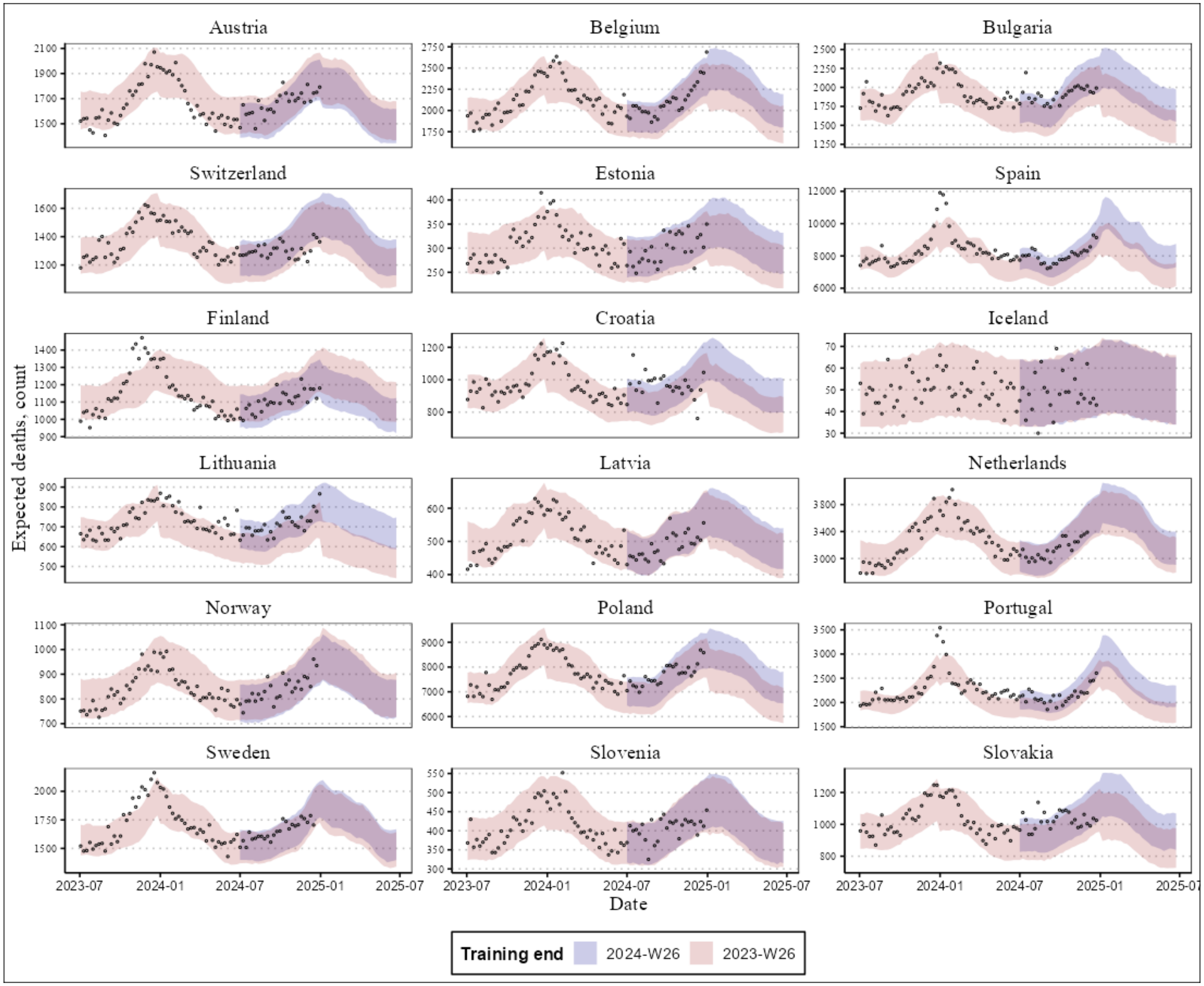

TextS5-F1. GAM model projections of national expected mortality (95% prediction interval) using data observed through 2023-W26 (red) and 2024-W26 (blue). Data points show observed mortality and are out-of-sample for '2023-W26' projections.

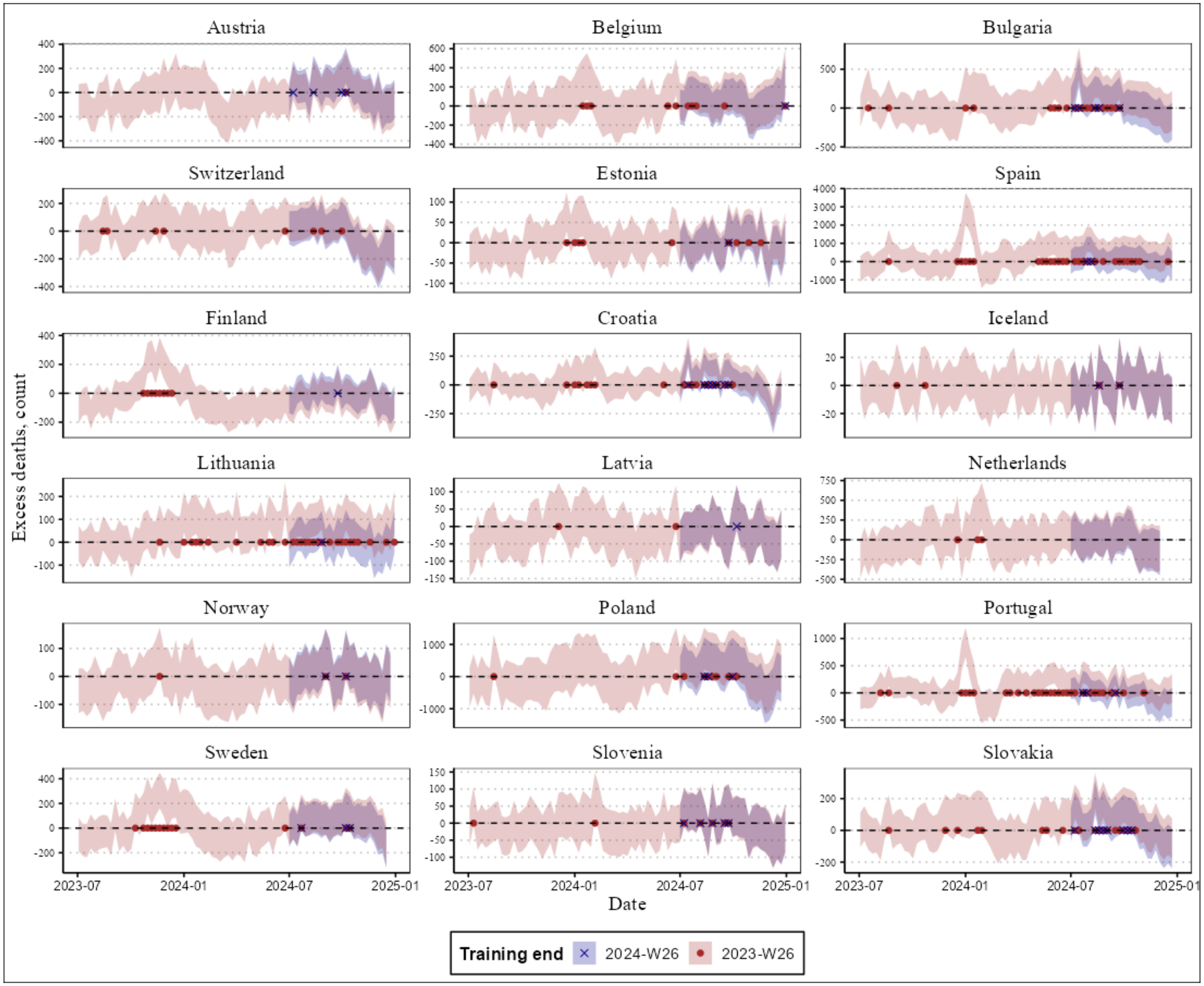

TextS5-F2. GAM estimated national excess mortality (95% PI) using data observed through 2023-W26 (red) and 2024-W26 (blue). Data points on the *y=0* line represent statistically significant excess mortality when trained on mortality through 2023-W26 ('o') or 2024-W26 ('x').

References

1. Cleveland RB, Cleveland WS, McRae JE, Terpenning I. STL: A seasonal-trend decomposition procedure based on loess. J Off Stat. 1990;6:3-73.
2. Hyndman R AG, Bergmeir C, Caceres G, Chhay L, O'Hara-Wild M, Petropoulos F, Razbash S, Wang E, Yasmeen F. forecast: Forecasting functions for time series and linear models. 8.20 ed2023.
3. Serfling RE. Methods for current statistical analysis of excess pneumonia-influenza deaths. Public health reports. 1963;78(6):494.
4. Muggeo VM. Segmented: an R package to fit regression models with broken-line relationships. R news. 2008;8(1):20-5.
5. Muggeo VM. Testing with a nuisance parameter present only under the alternative: a score-based approach with application to segmented modelling. Journal of Statistical Computation and Simulation. 2016;86(15):3059-67.
6. Muggeo VM. Selecting number of breakpoints in segmented regression: implementation in the R package segmented. Technical report. 2020.
7. Bosse NI, Gruson H, Cori A, van Leeuwen E, Funk S, Abbott S. Evaluating forecasts with scoringutils in R. arXiv preprint arXiv:220507090. 2022.
8. Bracher J, Ray EL, Gneiting T, Reich NG. Evaluating epidemic forecasts in an interval format. PLoS computational biology. 2021;17(2):e1008618.
9. Gneiting T, Raftery AE. Strictly proper scoring rules, prediction, and estimation. Journal of the American statistical Association. 2007;102(477):359-78.
10. Cramer EY, Ray EL, Lopez VK, Bracher J, Brennen A, Castro Rivadeneira AJ, et al. Evaluation of individual and ensemble probabilistic forecasts of COVID-19 mortality in the United States. Proceedings of the National Academy of Sciences. 2022;119(15):e2113561119.
11. EuroMOMO. What is a Z-score 2025 [Available from: https://www.euromomo.eu/how-it-works/what-is-a-z-score.
12. EuroMOMO. Methods. 2025 [Available from: https://www.euromomo.eu/how-it-works/methods.
13. EuroMOMO. Sample of the series used to fit the model. 2025 [Available from: https://www.euromomo.eu/how-it-works/methods#sample-of-the-series-used-to-fit-the-model.
14. EuroMOMO. What is the expected mortality baseline. 2025 [Available from: https://www.euromomo.eu/how-it-works/frequently-asked-questions#what-is-the-expected-mortality-baseline.
15. EuroMOMO (euromomo.eu) 2025 2025 [Available from: euromomo.eu.

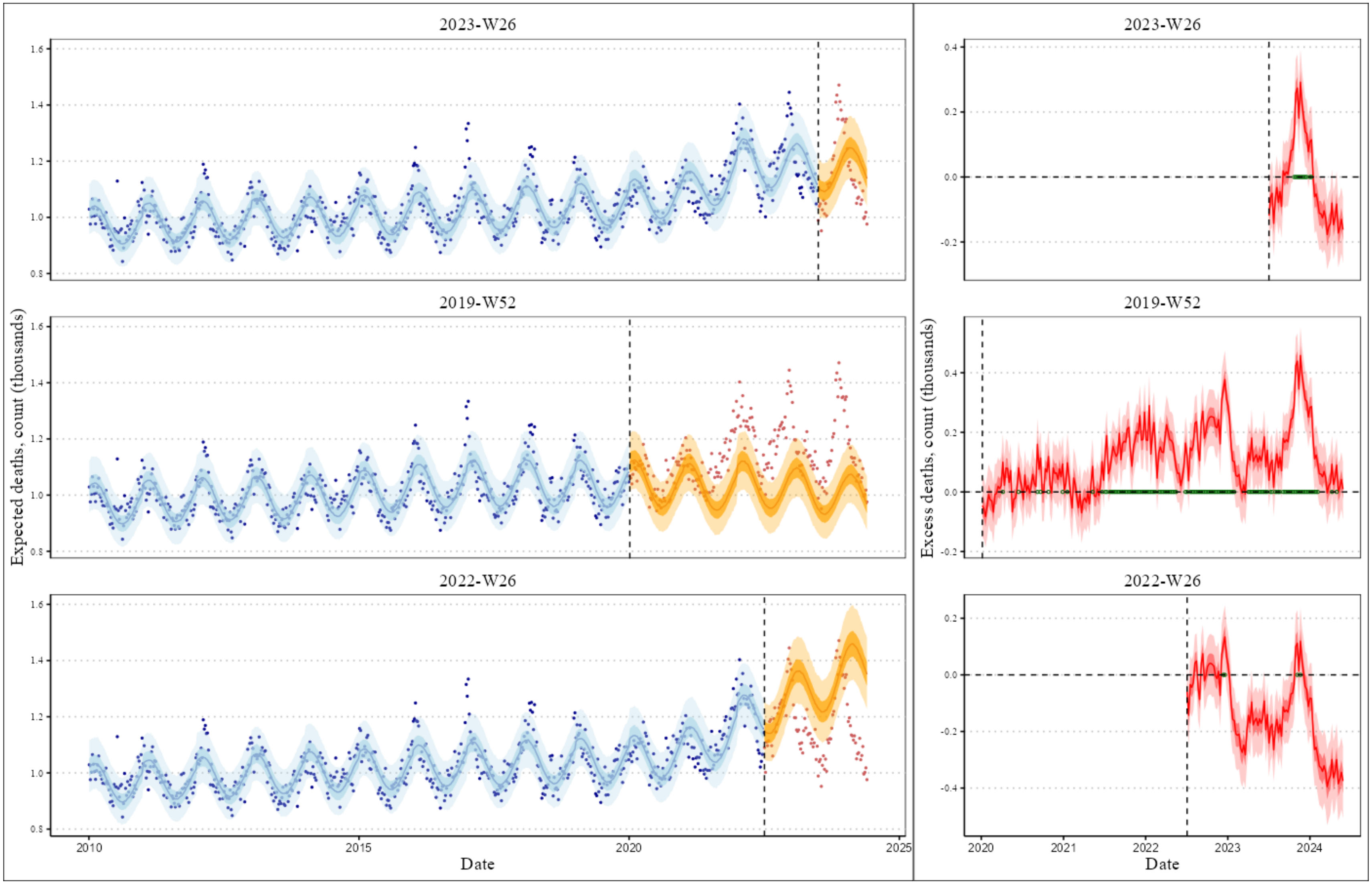

Supplementary Figure S1. GLM model fit (blue), predicted expected (orange) and excess deaths (red) in Finland, using observed mortality through 2023-W26 (*top*), 2019-W52 (*center*) and 2022-W26. Center darker band shows interquartile range (0.25-0.75 quantiles) and outer band shows 95% prediction interval. Data points show reported mortality. Weeks with statistically significant excess deaths are indicated with a '*' on *y*=0 line (right).

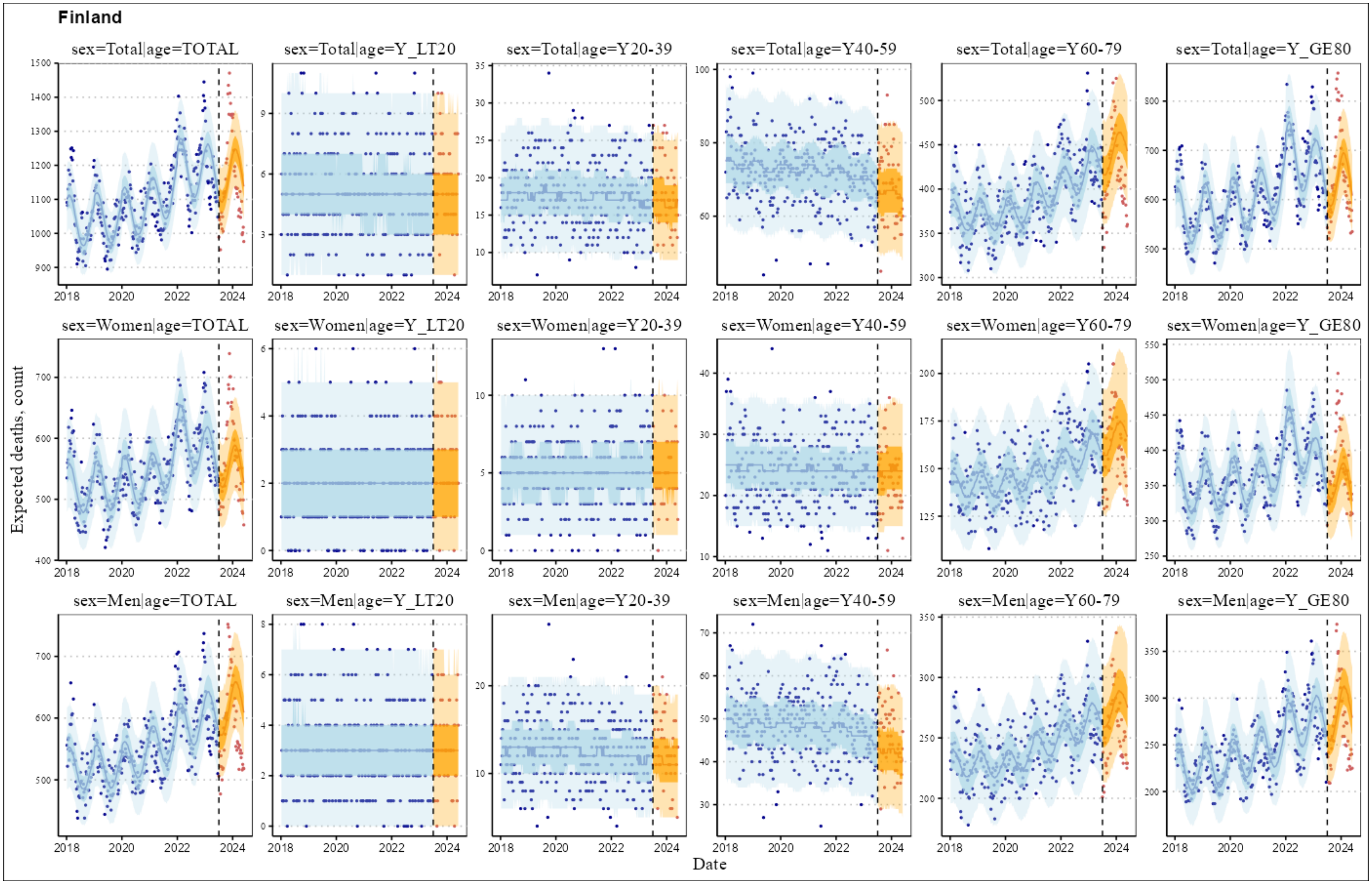

Supplementary Figure S2. GLM model fit (blue) and predicted expected deaths (orange) in Finland for different sex (rows) age groups (columns), using observed mortality through 2023-W26. Center darker band shows interquartile range (0.25-0.75 quantiles) and outer band shows 95% prediction interval. Data points show reported mortality; only observations available through 2023-W26 (blue) were used to train the model.

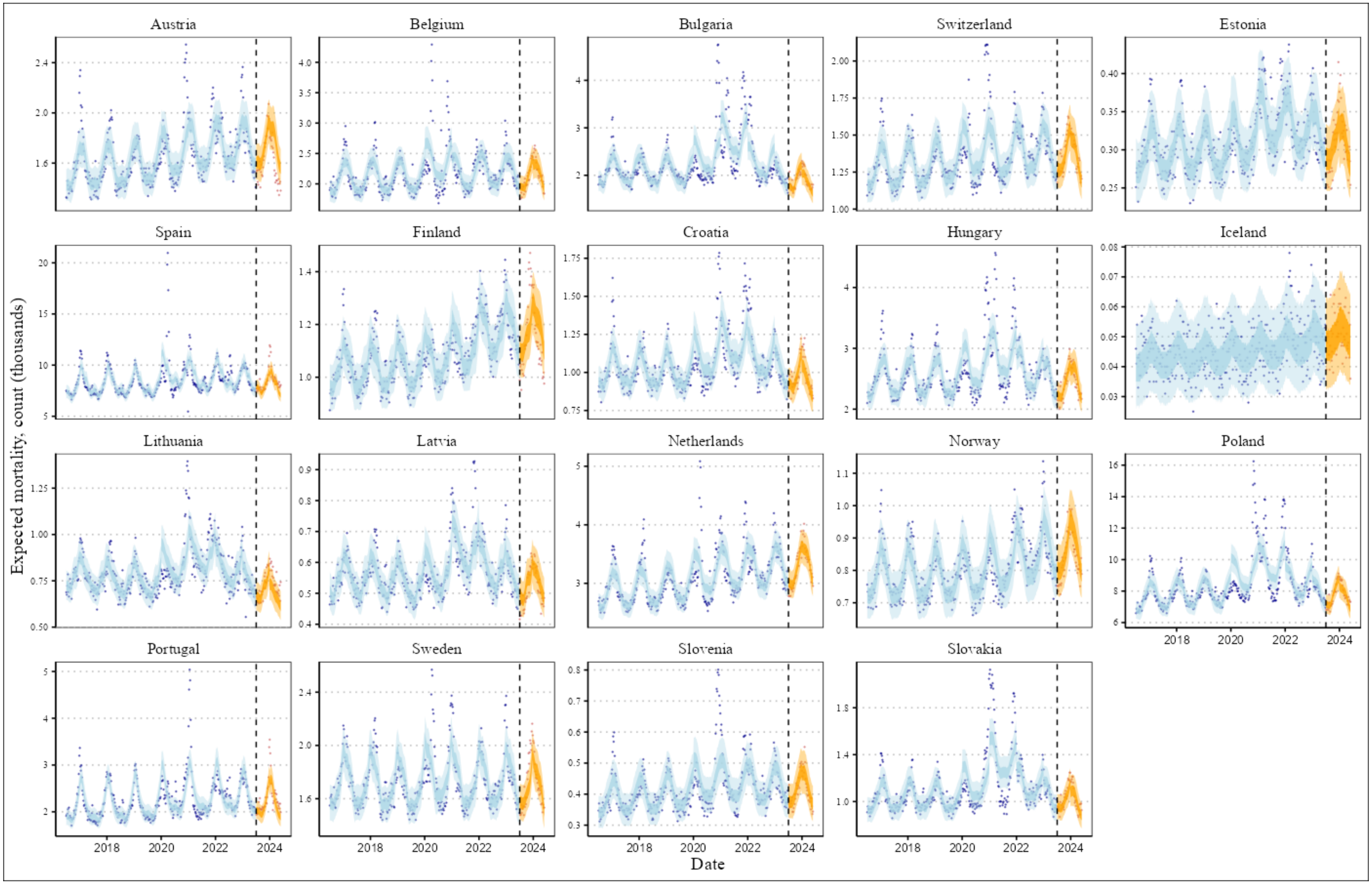

Supplementary Figure S3. GAM model fit (blue) and predicted expected deaths (orange) in multiple countries in Europe, using observed mortality through 2023-W26. Center darker band shows interquartile range (0.25-0.75 quantiles) and outer band shows 95% prediction interval. Data points show reported mortality.

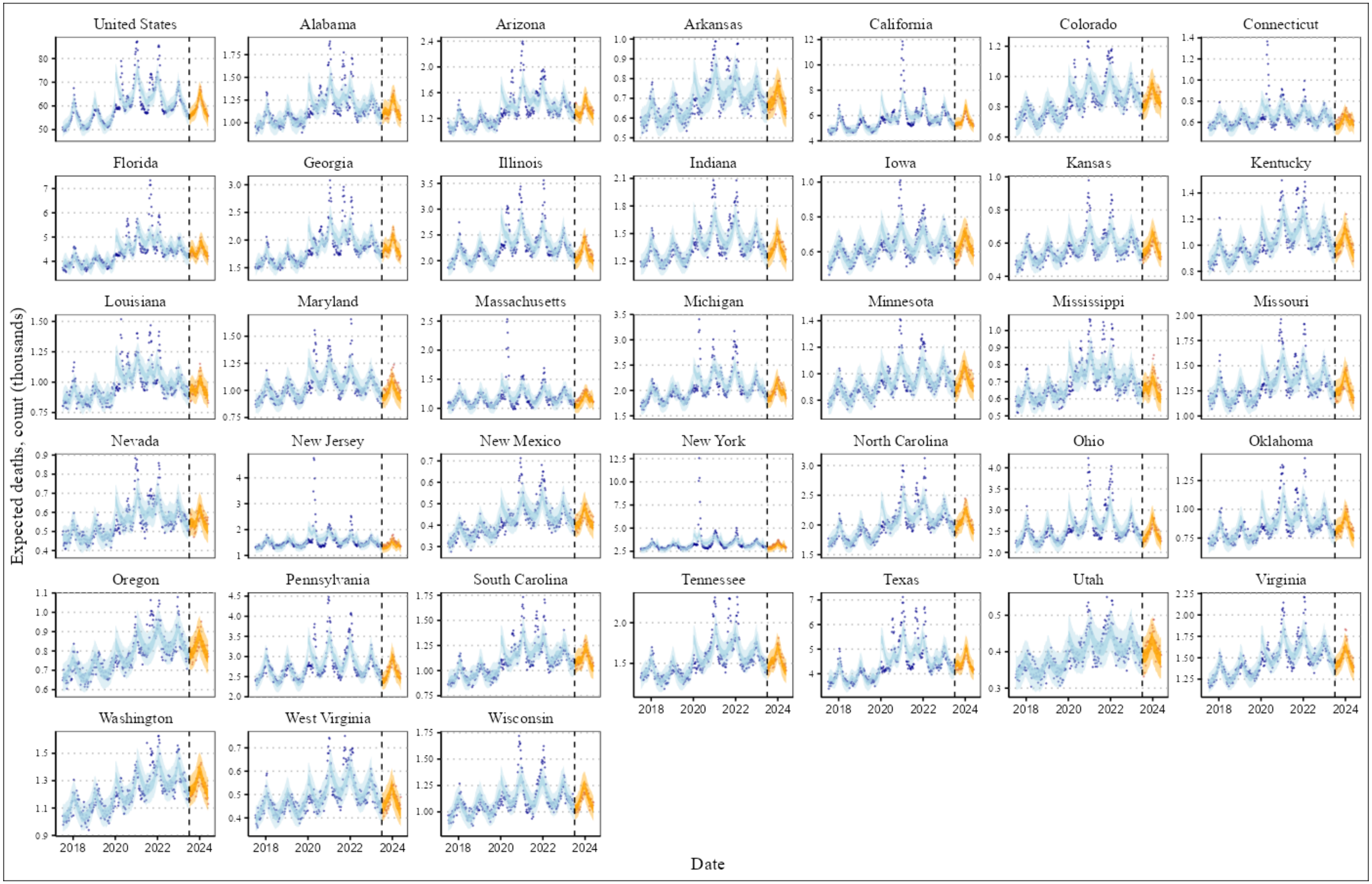

Supplementary Figure S4. GAM model fit (blue) and predicted expected deaths (orange) in the United States and multiple states, using observed mortality through 2023-W26. Center darker band shows interquartile range (0.25-0.75 quantiles) and outer band shows 95% prediction interval. Data points show reported mortality. States with substantial suppressed counts are not shown.

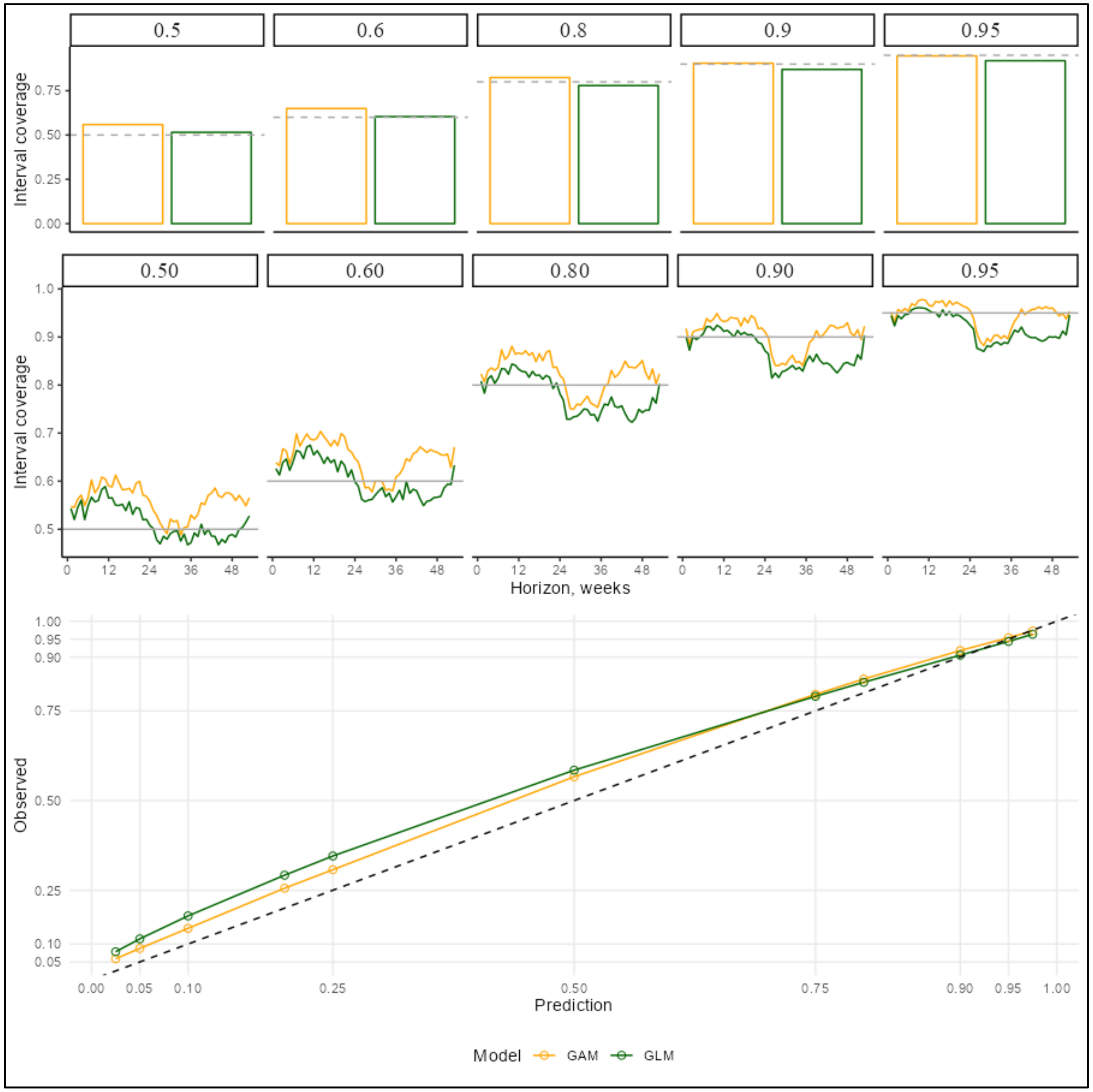

Supplementary Figure S5. Interval coverage for the two models across all countries overall (top), at different projected weeks (center) and confidence levels**.**

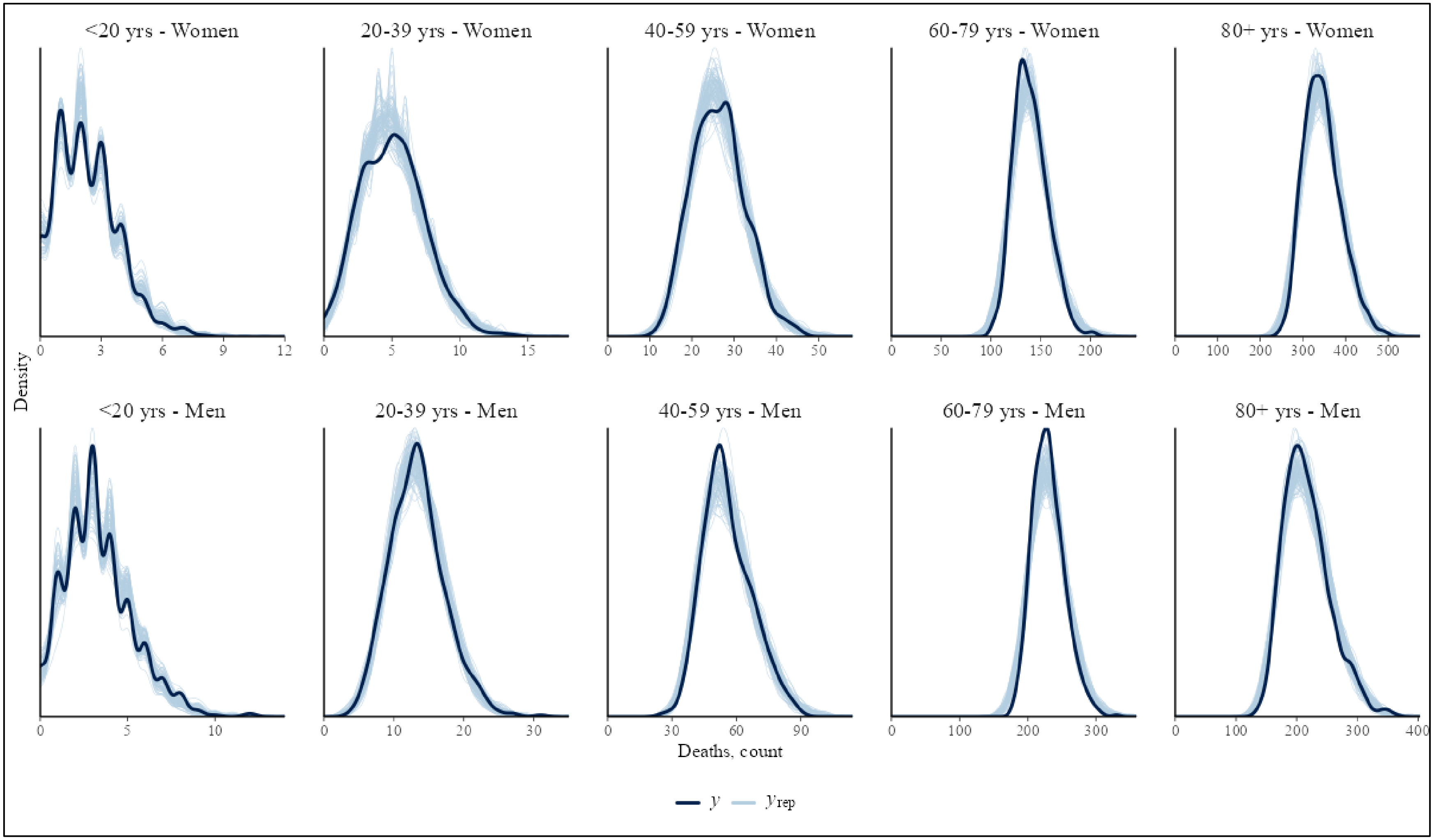

Supplementary Figure S6-a. Comparison of the empirical distribution of the data (*y)* to the distributions of simulated data (*yrep)* from the posterior predictive distribution of the **GAM** model fit to mortality observed in Finland through 2023-W26, by age group and sex of the decedent. Note that the range of the x-axis varies across panels.

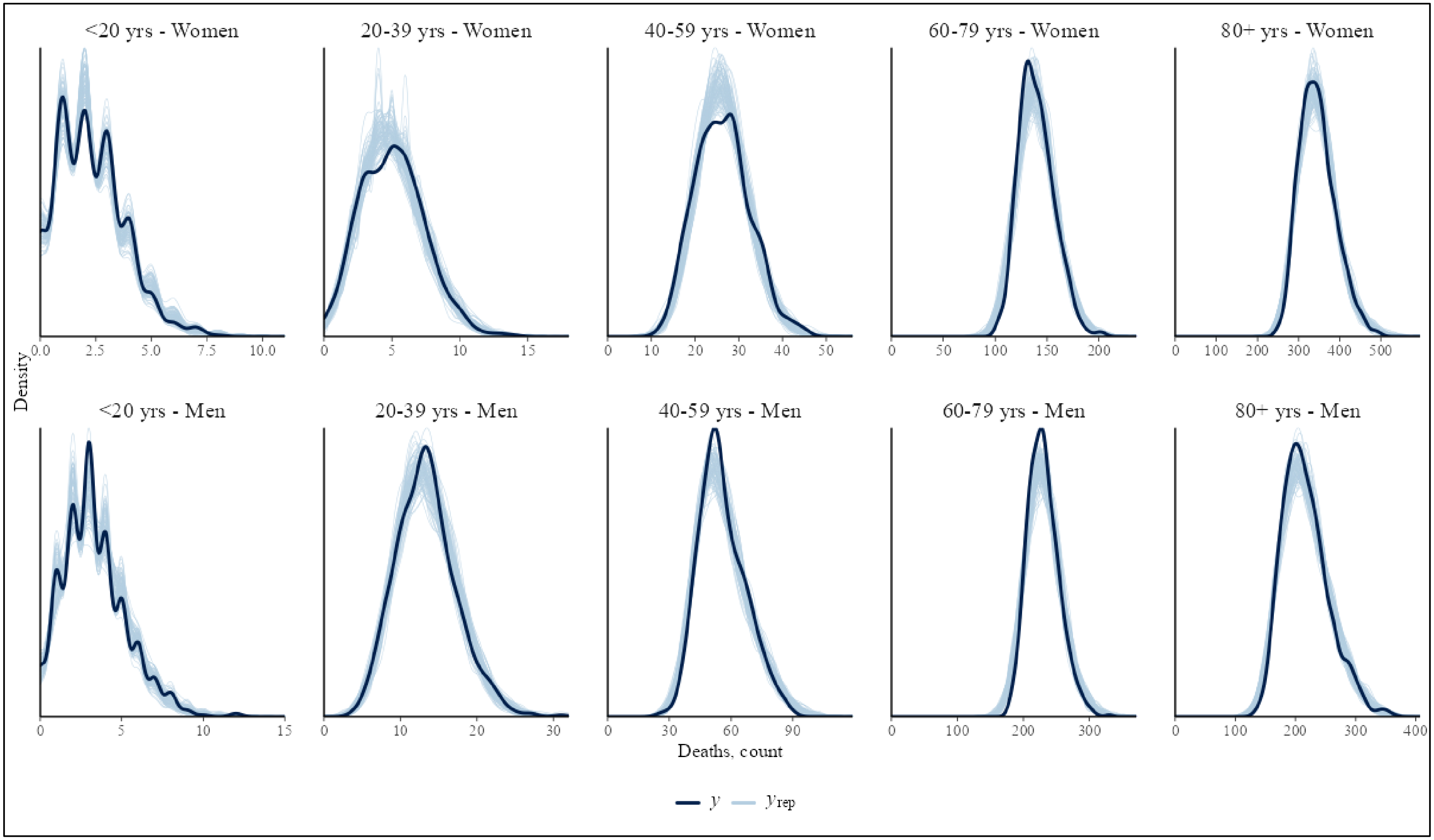

Supplementary Figure S6-b. Comparison of the empirical distribution of the data *(y)* to the distributions of simulated data (*yrep)* from the posterior predictive distribution of the **GLM** model fit to mortality observed in Finland through 2023-W26, by age group and sex of the decedent. Note that the range of the x-axis varies across panels.

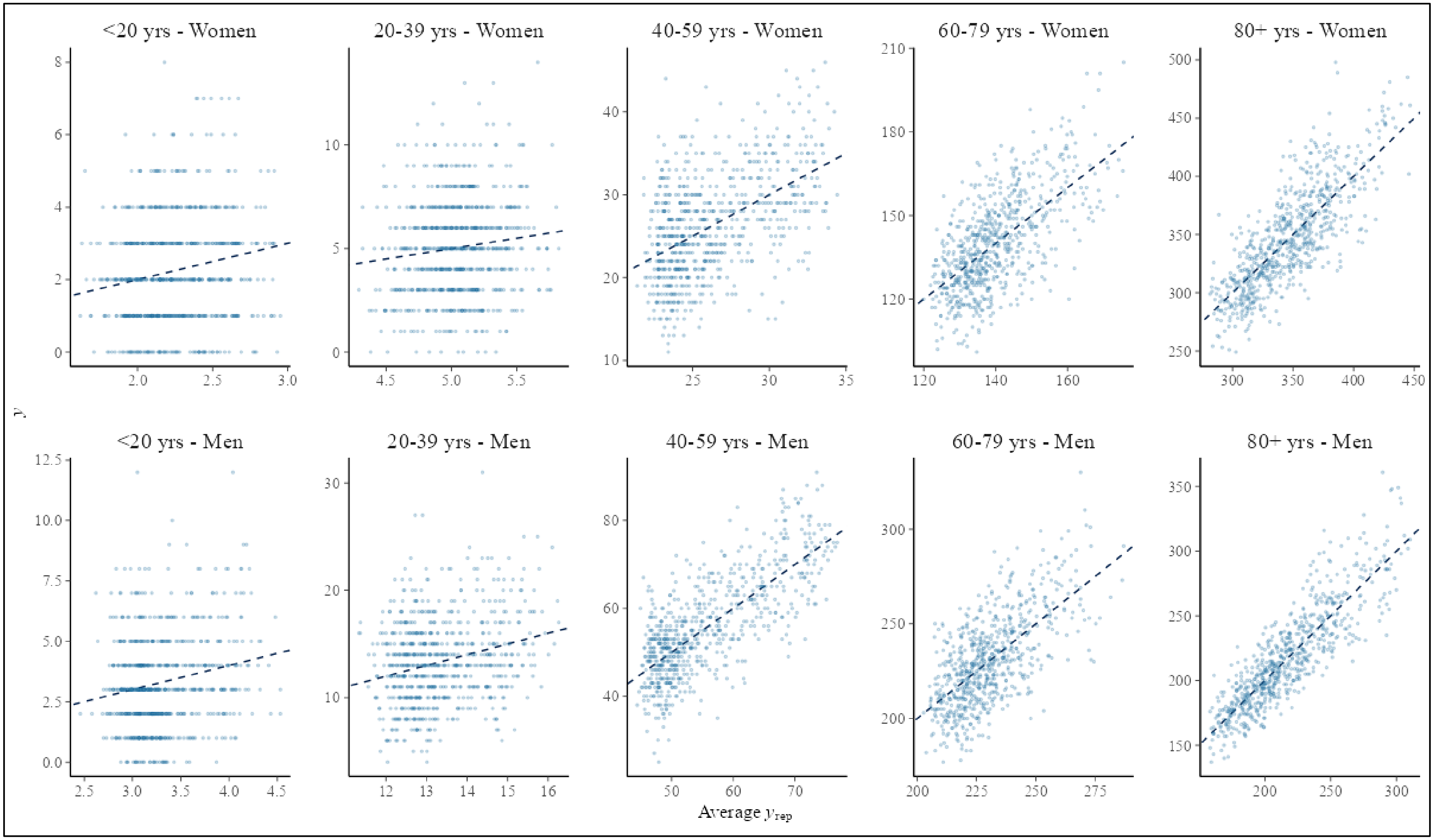

Supplementary Figure S7-a. Scatter plot of the observed data *(y)* vs average of simulated data *(x-axis)* from the posterior predictive distribution of the **GAM** model fit to mortality observed in Finland through 2023-W26, by age group and sex of the decedent. Note that the range of the x-axis varies across panels.

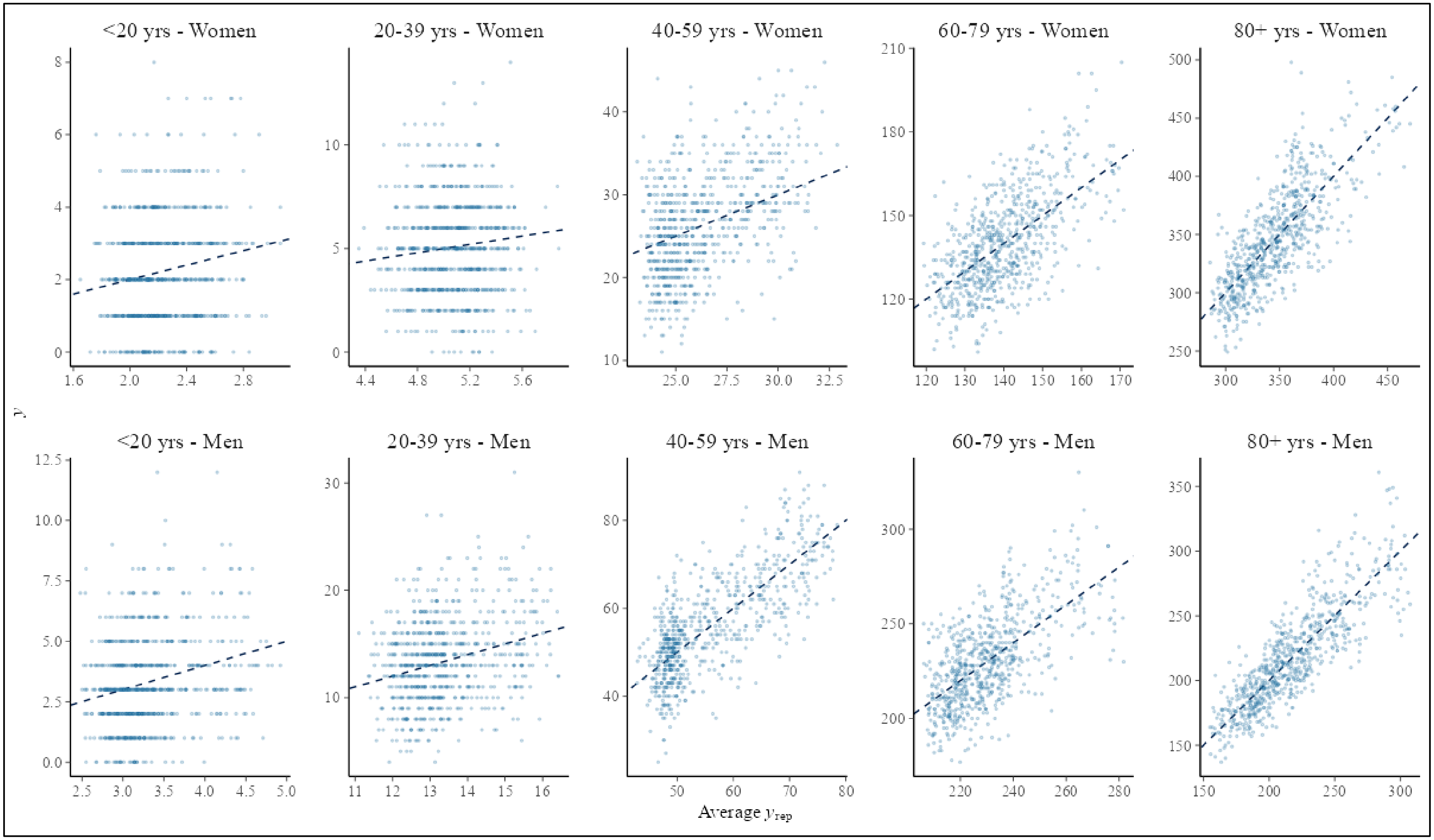

Supplementary Figure S7-b. Scatter plot of the observed data *(y)* vs average of simulated data (*x-axis)* from the posterior predictive distribution of the **GLM** model fit to mortality observed in Finland through 2023-W26, by age group and sex of the decedent. Note that the range of the x-axis varies across panels.

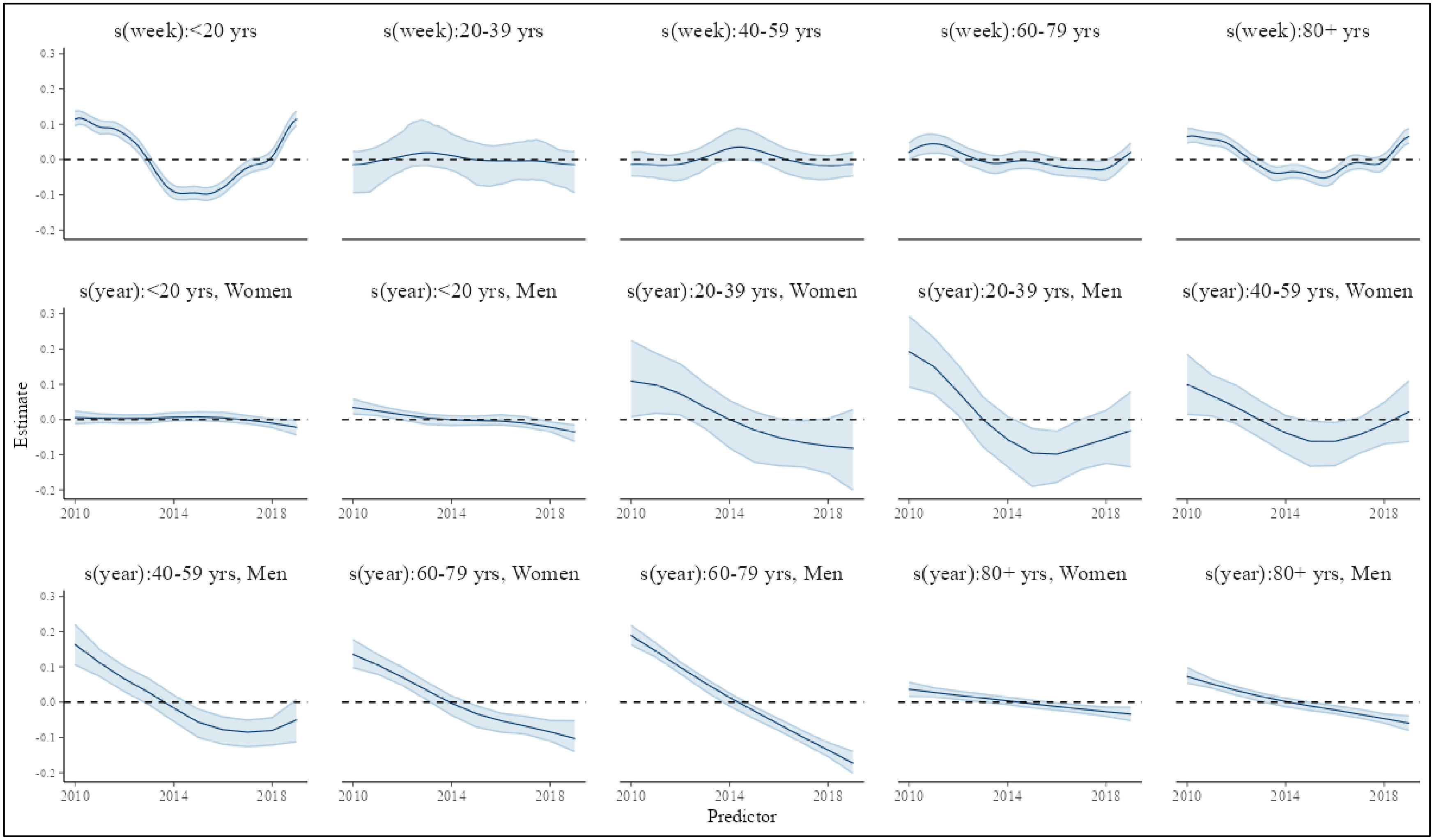

Supplementary Figure S8-a. GAM model estimated smoothing functions for weekly seasonality (top row) and annual trend using mortality observed in Finland through **2019-W52**. Weekly seasonality term stratified by age (*<20 yrs, 20-39 yrs, 40-59 yrs, 60-79 yrs, 80+ yrs*) and annual trend by age and sex (*Men, Women*).

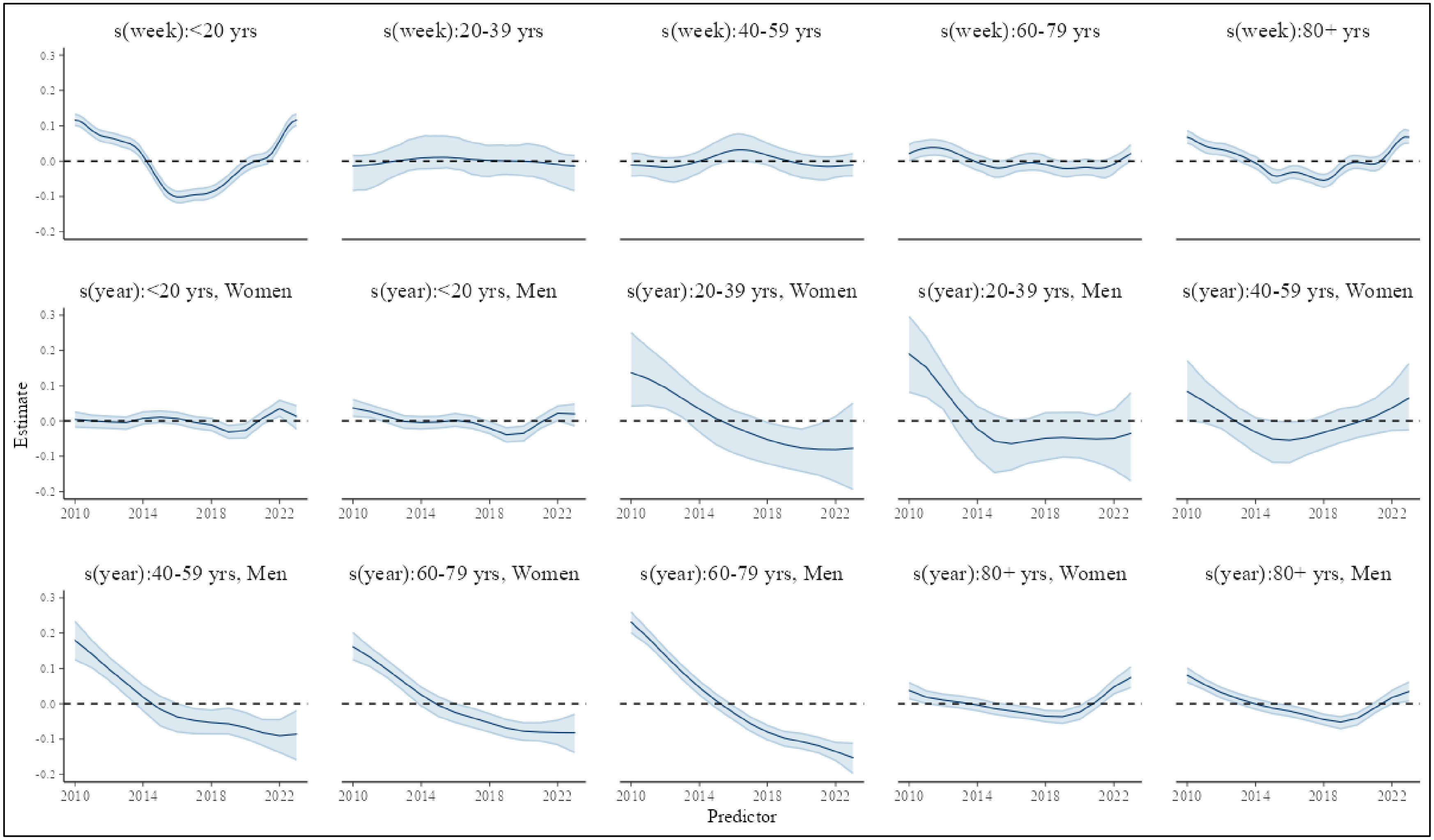

Supplementary Figure S8-b. GAM model estimated smoothing functions for weekly seasonality (top row) and annual trend using mortality observed in Finland through **2023-W26**. Weekly seasonality term stratified by age (*<20 yrs, 20-39 yrs, 40-59 yrs, 60-79yrs, 80+ yrs*) and annual trend by age and sex (*Men, Women*).

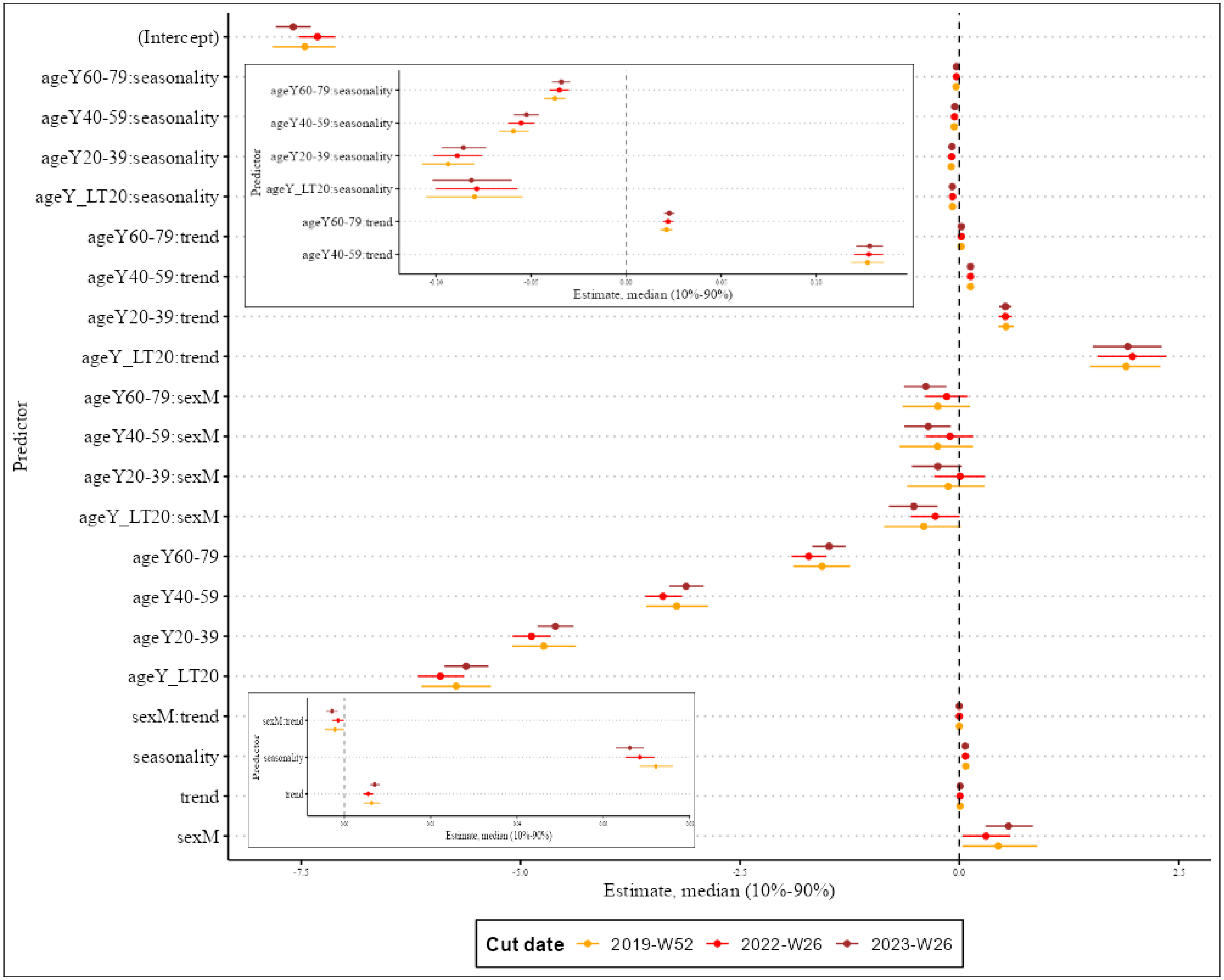

Supplementary Figure S9. Median (10%-90% credible interval) estimates for GLM model fit to mortality observed in Finland through 2019-W52 (orange), 2022-W26 (red), and 2023-W26. Insets show select predictors on a smaller effects scale. ':' indicates an interaction term. 80+ year old decedents and corresponding interaction terms were set as reference.
