# Supplementary material for: Real-time monitoring of excess mortality under a new endemic regime
Source: Euro Surveill. 2025 Jun 26;30(25):2400753. doi: 10.2807/1560-7917.ES.2025.30.25.2400753 (PMC12207195; doi:10.2807/1560-7917.ES.2025.30.25.2400753)
Supplement: Supplement_United_States [file 24-00753_KANDULA_Supplement_United_States.pdf]

This supplementary material is hosted by Eurosurveillance as supporting information alongside the article Real-time monitoring of excess mortality under a new endemic regime *on behalf of the authors who remain responsible for the accuracy and appropriateness of the content. The same standards for ethics, copyright, attributions and permissions as for the article apply. Eurosurveillance is not responsible for the maintenance of any links or email addresses provided therein.*

Supplementary text for mortality in United States.

Summary findings

Due to space restrictions, in the main text we focused only on mortality estimates for countries in Europe, and here we report summary results for the United States, nationally and in select 37 states. Nationally, a large proportion of the excess deaths were estimated to have occurred before summer of 2022 (Figure US-F1, middle row). The models were responsive to inclusion of mortality during parts of the acute pandemic period. For the most recent year modeled using mortality observed until 2023-W26 (Figure US-F1, top row), the expected mortality was consistent with observed mortality, and no significant excess deaths were estimated.

Age-stratified estimates indicate that the GAM was able to approximate the trend as well as the varying degree of seasonality in each age group (Figure US-F2). The continuation of slight elevation in overall mortality relative to pre-pandemic level beyond summer of 2022, appears to be predominantly concentrated in the 75-84 year population (Figure US-F3, middle row). As noted in the main text, mortality data for US was available for a shorter historical period, which made a cross-validation exercise like the one reported for the European countries infeasible here.

For the period between 2023-W27 and 2024-W26, in nearly all 37 states (possible exception of Georgia, Texas) for which we were able to generate estimates mortality seems to have returned to pre-pandemic levels (Figure US-F4). This is inline with findings from Europe. However, Figure US-F5 indicates that during several weeks of winter of 2023-2024, there was a high probability of excess mortality in most states, relative to both pre-pandemic mortality as well as mortality seen through 2023-W26. Weekly state-level estimates are included in the supplementary dataset *US_Estimates* (xlsx file).

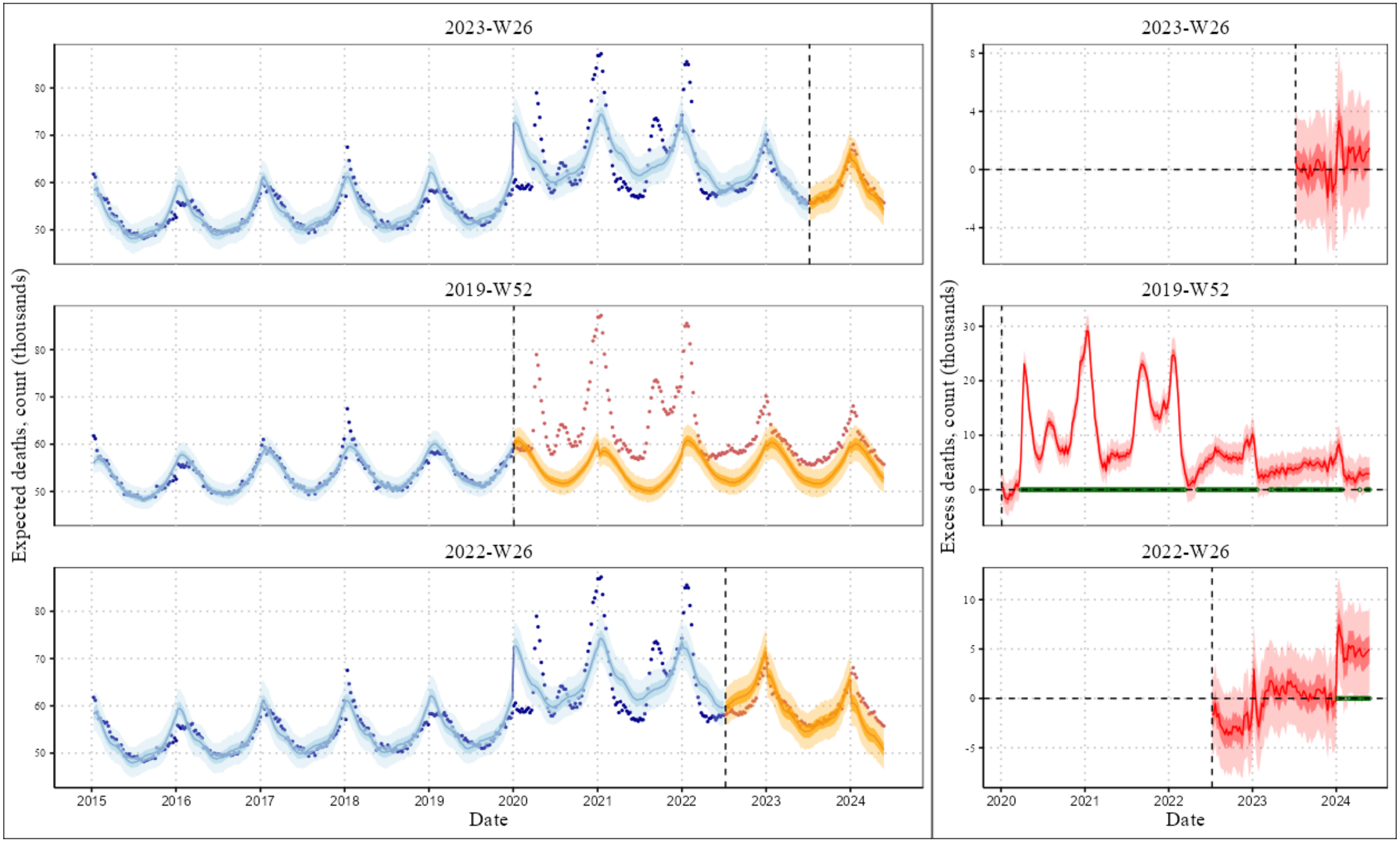

Figure US-F1. GAM model fit (blue), predicted expected deaths (orange) and excess deaths (red, right panels) in United States nationally, using observed mortality through 2023-W26 (*top*), 2019-W52 (*center*) and 2022-W26. Center band shows interquartile range (0.25-0.75), and outer band shows 95% PI. Data points show reported mortality. Weeks with statistically significant excess are indicated by a green point on *y*=0 line (right).

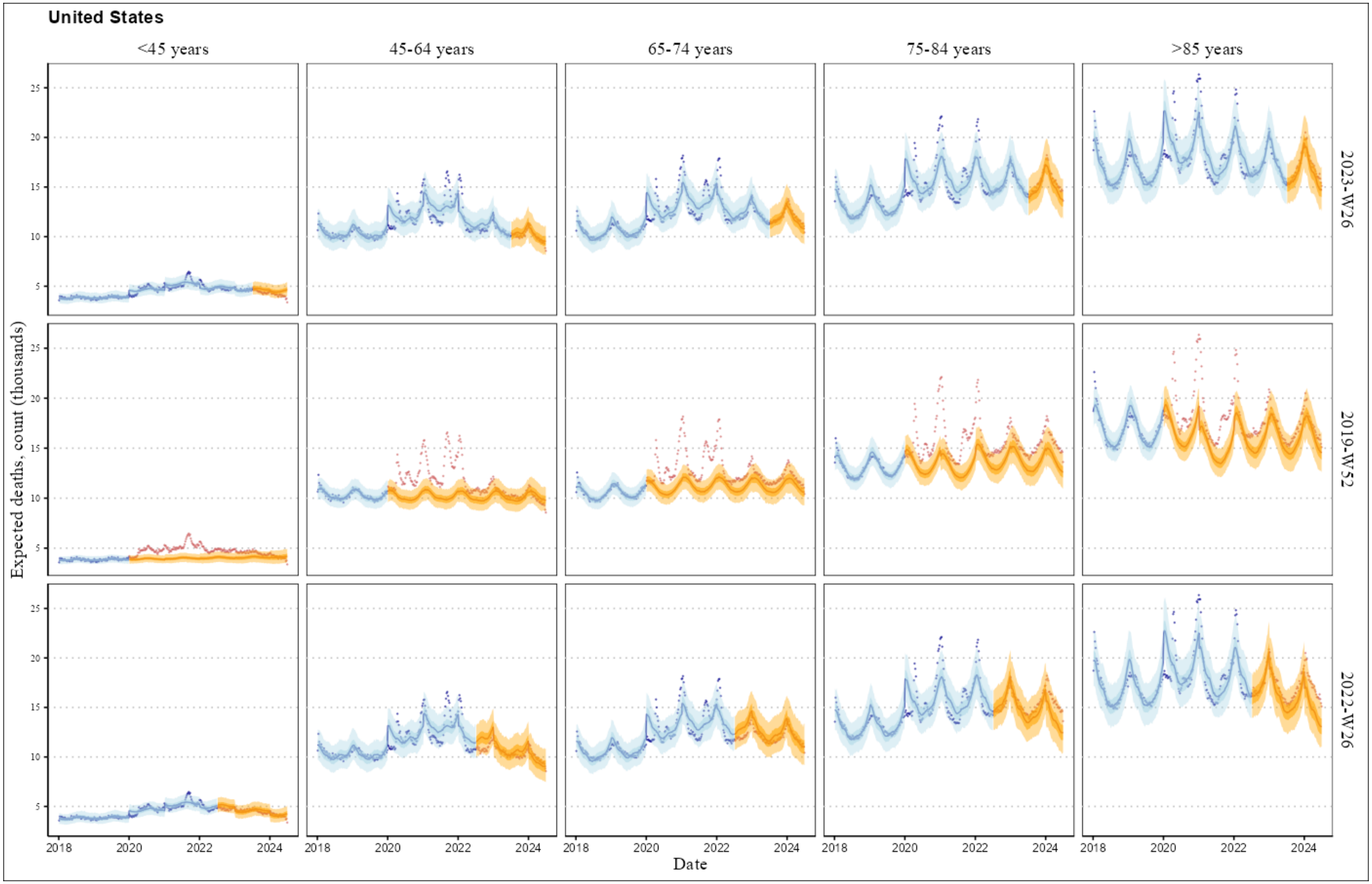

Figure US-F2. GAM model fit (blue), predicted expected deaths (orange) in United States stratified by age (columns), using observed mortality through 2023-W26 (*top*), 2019-W52 (*center*) and 2022-W26. Center band shows interquartile range (0.25-0.75), and outer band shows 95% PI. Data points show reported mortality.

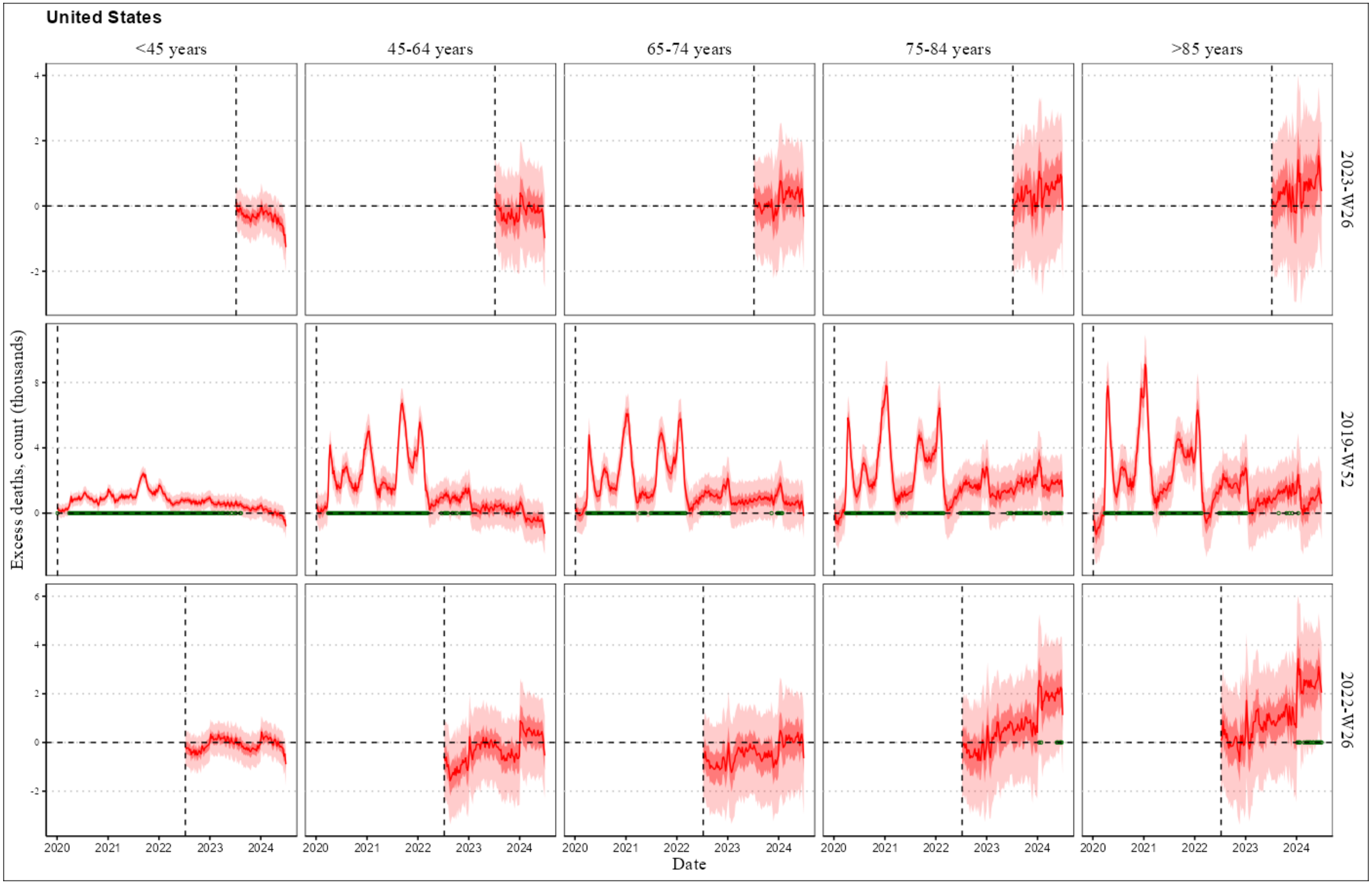

Figure US-F3. GAM model estimated excess deaths in United States stratified by age (columns) using observed mortality through 2023-W26 (*top*), 2019-W52 (*center*) and 2022-W26. Center band shows interquartile range (0.25-0.75), and outer band shows 95% PI. Weeks with statistically significant excess are indicated by a green point on *y*=0 line.

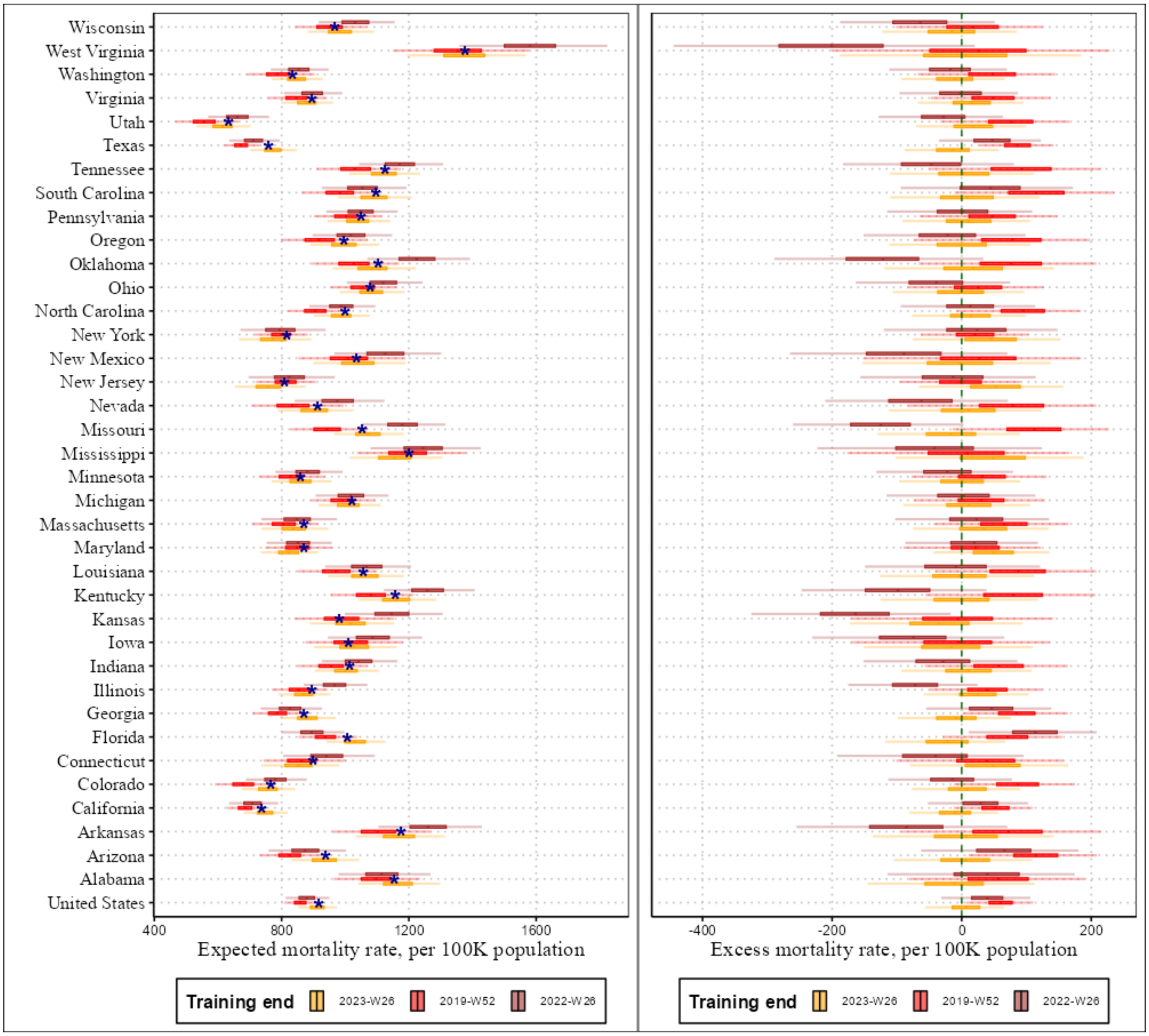

Figure US-F4. National and state-level estimates of expected (left) and excess mortality rates per 100,000 population between 2023-W27 and 2024-W26, with GAM model trained on pre-pandemic mortality (orange) and two periods of acute pandemic activity, up to 2022-W26 (red) and 2023-W26 (brown). '*' denotes observed mortality rate for the period. The lighter and darker bands show 95% and 50% prediction intervals, respectively.

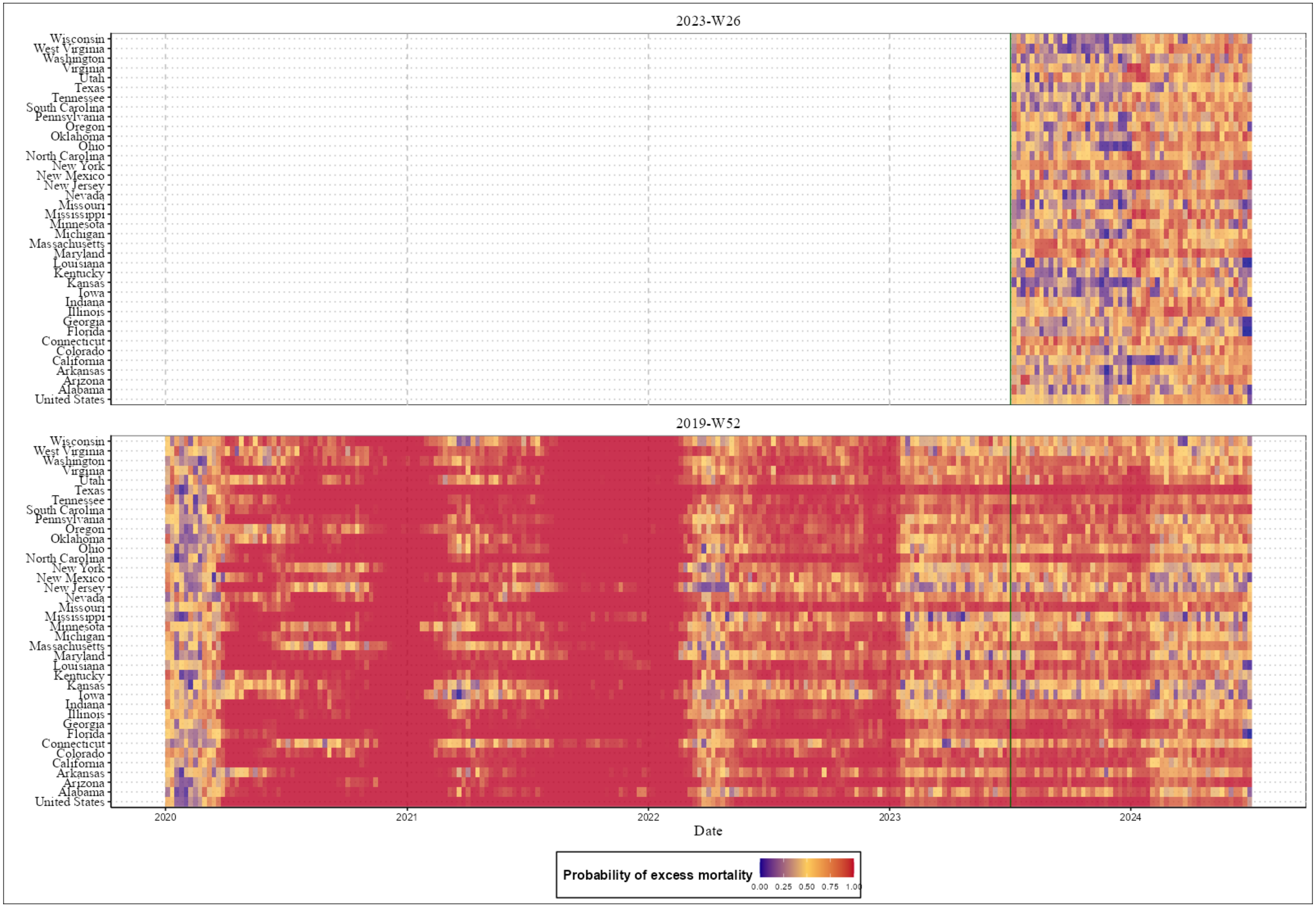

Figure US-F5. Probability of excess mortality in a state (y-axis) during a week (x-axis)**,** as estimated by the GAM model, using data available through 2019-W52 (top) and 2023-W26.
